# A Global Metabolic Map Defines the Effects of a Si-Based Biostimulant on Tomato Plants under Normal and Saline Conditions

**DOI:** 10.3390/metabo11120820

**Published:** 2021-11-30

**Authors:** Kekeletso H. Chele, Paul Steenkamp, Lizelle A. Piater, Ian A. Dubery, Johan Huyser, Fidele Tugizimana

**Affiliations:** 1 Department of Biochemistry, University of Johannesburg, Auckland Park, Johannesburg 2006, South Africa; ckekeletso@gmail.com (K.H.C.); psteenkamp@uj.ac.za (P.S.); lpiater@uj.ac.za (L.A.P.); idubery@uj.ac.za (I.A.D.); 2 International Research and Development Division, Omnia Group, Ltd., Johannesburg 2021, South Africa; Johan.Huyser@omnia.co.za

**Keywords:** biostimulants, GNPS, metabolomics, molecular networking, salt stress, Si-biostimulant, stress tolerance, tomato plants

## Abstract

The ongoing unpredictability of climate changes is exponentially exerting a negative impact on crop production, further aggravating detrimental abiotic stress effects. Several research studies have been focused on the genetic modification of crop plants to achieve more crop resilience against such stress factors; however, there has been a paradigm shift in modern agriculture focusing on more organic, eco-friendly and long-lasting systems to improve crop yield. As such, extensive research into the use of microbial and nonmicrobial biostimulants has been at the core of agricultural studies to improve crop growth and development, as well as to attain tolerance against several biotic and abiotic stresses. However, the molecular mechanisms underlying the biostimulant activity remain enigmatic. Thus, this study is a liquid chromatography-mass spectrometry (LC-MS)-based untargeted metabolomics approach to unravel the hypothetical biochemical framework underlying effects of a nonmicrobial biostimulant (a silicon-based formulation) on tomato plants (*Solanum lycopersium*) under salinity stress conditions. This metabolomics study postulates that Si-based biostimulants could alleviate salinity stress in tomato plants through modulation of the primary metabolism involving changes in the tricarboxylic acid cycle, fatty acid and numerous amino acid biosynthesis pathways, with further reprogramming of several secondary metabolism pathways such as the phenylpropanoid pathway, flavonoid biosynthesis pathways including flavone and flavanol biosynthesis. Thus, the postulated hypothetical framework, describing biostimulant-induced metabolic events in tomato plants, provides actionable knowledge necessary for industries and farmers to, confidently and innovatively, explore, design, and fully implement Si-based formulations and strategies into agronomic practices for sustainable agriculture and food production.

## 1. Introduction

The severity of salt stress has been predicted to escalate, with over 50% of arable land estimated to be heavily salinised by 2050, posing a great threat to food security [1]. Salinity has been described as an accumulation of salts in soil, primarily sodium (Na^+^) and chloride (Cl^−^) ions. Excessive amounts of salt ions in the rhizosphere could occur naturally from processes such as mineral weathering and gradual withdrawal from the ocean, or because of irrigation malpractices and overuse of fertilisers [2]. Consequently, salt stress affects multiple vital processes of plants, leading to several modifications in the physiological and metabolic processes, both of which are dependent on the duration and severity of the stress. These changes manifest in two salt stress-induced phases: the earlier phase (osmotic stress) and the late phase (ionic stress) [3]. Plant responses to environmental stresses are multi-layered and highly regulated complex phenomena, which involve reprogramming of several cellular, molecular and physiological networks [4]. In addition to nuances in the basis of salt tolerance in plants, some of the key cellular and molecular events that fundamentally define the plant responses to salinity stress include ion homeostasis, signalling (reactive oxygen species (ROS), nitric oxide (NO), Ca^2+^, hormones), and metabolic remodelling [5,6]. Regardless of the nature of the plant, be it glycophytes or halophytes, both cannot tolerate excessive amounts of salt in the cytoplasm [3]. Under salt stress conditions, plants eliminate excess salt ions actively via either transportation into the vacuole or seclusion into other parts of the plant which are eventually sacrificed to protect the plant from stress.

Salt stress inhibits several metabolic pathways and enzymatic activities, resulting in accumulation of ROS which have roles as second messengers in stress signalling [7,8]. In addition to second messengers (ROS and Ca^2+^), phytohormones including abscisic acid (ABA), jasmonic acid (JA), ethylene and gibberellins play significant signalling roles in response to salt stress, ultimately stimulating modulation of biochemical and physiological processes involved in plant defences [1,9]. Such signalling cascades are the frontier of plant responses to salt stress, ultimately leading to alterations in gene and protein expression for synthesis of stress response-related molecules. Such metabolic reprogramming involve reconfiguration of both the primary and secondary metabolic pathways involving synthesis of stress responsive metabolites including amino acids, organic acids, polyamines and phenolic compounds [10,11,12,13]. As sessile organisms, plants have naturally and evolutionarily developed responses against abiotic stresses, however, the extremity and persistence of such stresses can often lead to huge crop losses. As a result, the main objective of modern agriculture is to improve crop productivity even in unfavourable salinity conditions through incorporation of innovative and eco-friendly approaches, among which is the use of biostimulants [14].

A biostimulant is conceptually described as any substance or microorganism that is not a nutrient, pesticide or any of the soil improvers, but can promote the health and growth of a plant through the induction of natural biological processes [15,16,17]. Plant biostimulants can be classified into two main groups including microbial and non-microbial biostimulants. Observed and elucidated effects of biostimulants include increasing the rate at which the plant absorbs and assimilates the nutrients, and improving the quality traits of crop plants [15,18]. Furthermore, studies have shown that the application of biostimulants can enhance plant defence mechanisms, resistance and tolerance against biotic and abiotic stresses [19,20]. Si (Si)-based formulations hold potential for improving plant growth and tolerance under salt stress conditions [21,22]. According to research, the Silicon-mediated salt stress alleviation mechanisms include (i) enhancement of water retention via curbing of transpiration, (ii) maintenance of water content, thus reducing salt-induced osmotic stress, (iii) regulation of gene expression relating to plant hormones, compatible solutes, antioxidant enzymes and ion pumps biosynthesis and (iv) alleviation of ion toxicity [23].

Although studies have suggested the abovementioned mechanisms for Si-mediated salinity stress alleviation, the limited fundamental knowledgebase of the modes of action of many biostimulant products is one of the gaps that require scientific attention. The elucidation of the biological basis of biostimulant function, and a broad mechanism of action at a molecular level, is a prerequisite for the development of a scientifically-based biostimulant industry, leading to an effective exploration and application of biostimulants in agriculture [16,24]. Systems biology approaches, such as metabolomics—a multidisciplinary science involving fourth industrial revolution (4IR) technologies—offer an unique opportunity to understand the modes of actions of plant biostimulants, revealing key metabolic landscapes that define plant responses to biostimulant applications [25,26,27]. Such insights would facilitate the development of effective biostimulant formulations contributing towards sustainable agriculture and food security. Thus, the work presented here is a metabolomics study to unravel a hypothetical framework that describes underlying biochemical and molecular events of the effects of a Si-based biostimulant on tomato plants under salinity stress conditions.

## 2. Results and Discussion

The measurement of the metabolome provides functional readouts and mechanistic insights of the biological system under consideration [28]. Hence, this global and data-driven (*omics*) approach aims to generate predictive models that intelligently grasp and describe the metabolic landscapes explaining (at a molecular level) the stress-alleviation of Si-based biostimulants (Section 3). For epistemological articulation of the findings from this study, results are presented and discussed into three main subsections: (i) the metabolome coverage and metabolic pathway context; and biological meanings of the biostimulant-induced alterations in tomato (ii) primary and (iii) secondary metabolism, under salt stress conditions.

### 2.1. Metabolome Coverage and Metabolic Pathway/Network Context

Hydromethanolic extracts of biostimulant-treated and tomato leaves (under both normal and salt stress conditions) were analysed on a reversed phase liquid chromatography coupled to a high-resolution mass spectrometry (LC-ESI-QTOF-MS) system. Chromatographically, the tomato samples contained a wide range of metabolites of different polarities, with treatment-related differential changes (Figure S1). These observed differential chromatographic fingerprints point to treatment-induced changes in the metabolism of tomato plants. Thus, to take an inventory of the measured metabolomes, the LC-MS/MS data were submitted to mass spectral molecular networking through the global natural product social molecular networking (GNPS) ecosystem (https://gnps.ucsd.edu (accessed on 21 June 2021)) [29] (Table S1). Mass spectral molecular networking enables a broad overview of molecular information that can be inferred from MS/MS data [30,31]. In molecular networking, all identical MS/MS spectra are merged giving a list of unique MS/MS spectra [32]. These are then subjected to spectral alignment allowing for spectral matching with offsets based on the precursor mass differences. Molecules generating similar MS/MS spectra are clustered due to similarities in their fragmentation patterns and are referred to as molecular families, i.e., structurally related. In addition, the MS/MS spectra are putatively annotated against reference spectra within the GNPS infrastructure [30,33].

**Figure 1:**
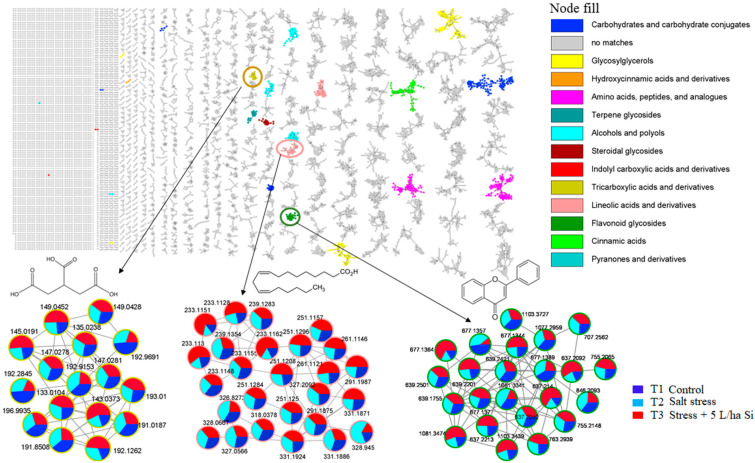
Molecular network of all detected ions using MS/MS data in the negative mode. Nodes from the selected clusters (tricarboxylic acids, linoleic acids, and flavonoids) are labelled with parent masses and displayed as pie charts representing distribution of ion intensities in the non-stressed controls (T1: dark blue), salt stressed (T2: light blue) and Si treated salt stressed samples (T3: red).

**Figure 2:**
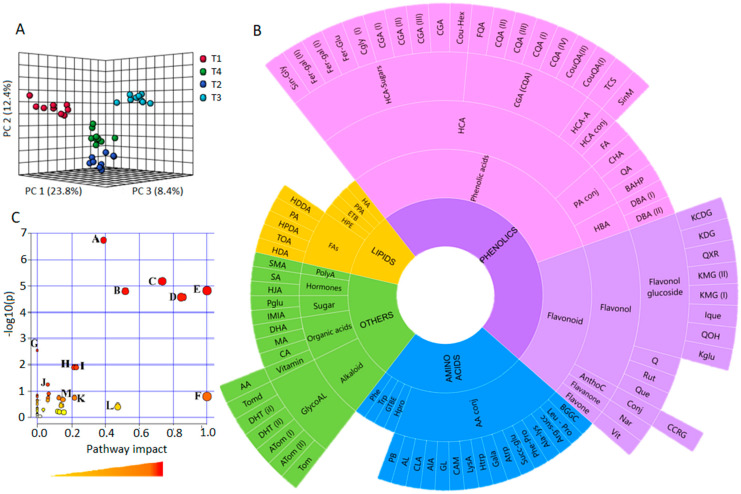
An overview of the metabolome coverage and metabolic changes associated with Si-biostimulant treatment of salt stressed plants. (**A**) The 3-dimensional PCA scores plot with 44.6% of 58.5% total explained variation between the non-stressed controls (T1: red), salt stressed (T2: dark blue), and the soil and foliar biostimulant treated stressed plants (T3: light blue and T4: green, respectively (**B**) A sunburst chart that summarises the overall metabolome covered in this study, displaying the main metabolite classes (amino acids, lipids, phenolics and others), their respective metabolites and conjugates. (**C**) The metabolome summary of pathway analysis shows the contribution of each pathway on biostimulant-induced metabolic changes. Node colour: significance of the pathway based on *p*-value (red is the most impactful). Node size: impact of each pathway, largest nodes represent pathways with the greatest impacts (See Table S3). Metabolite abbreviations are provided in Table S2.

This exploration of the collected ‘fragmentome’ therefore allows for the visual inspection of the chemical similarity between the annotated metabolites and the unknown molecules, hence expanding the metabolome coverage under scrutiny [34,35]. In this study, molecular networking of the metabolome in question revealed structurally related molecular families in tomato plants: the computed network consisted of 11,346 mass spectral nodes organized into 14 independent molecular families (Figure 1). The latter span a spectrum of primary metabolite classes including amino acids, tricarboxylic acids, and fatty acids and several secondary metabolite classes such as the terpenes, cinnamic acids and flavonoids. Furthermore, the computed molecular networks provided quantitative description of the measured metabolome by displaying nodes (ions) as pie charts to reflect the relative metabolic changes related to the effects of the Si-based biostimulant on tomato plants responding to salt stress conditions (Figure 1 and Figure 2). As infographically displayed in Figure 1, the selected clusters representing tricarboxylic acids and derivatives (A), linoleic acids and derivatives (B) and flavonoid glycosides (C), showed the relative quantification of respective ions under non-stressed, salt stressed and biostimulant treatment conditions. These metabolic changes point to roles of the altered metabolites in plant responses to salt stress, as discussed in detail in sections below.

To get an overview of the data, principal component analysis (PCA) was applied, summarising the variation in the data into a smaller number of latent components. The computed PCA models uncovered the overview structure of the data, exploratively revealing distinct treatment-related sample groupings, which include the non-stressed controls, salt stressed and Si-treated salt stressed sample groups (Figure 2A and Figure S2). These differential sample groupings evidently reflect an underlying biostimulant-induced metabolic reprograming of tomato plants under salt stress conditions, irrespective of the biostimulant concentration and the application area. For the biological interpretability of the data (i.e., evaluating and biologically explaining these PCA-revealed sample groupings), a classification model that also allows an identification of metabolic markers was then used. Classification can be done using linear ML methods such as PLS and OPLS or non-liner methods such as neural network (NN) and support vector machine (SVM) algorithms [25,36]. In this study, linear (supervised) ML methods, (O)PLS-DA (Figure S3A) were applied, with a perfect sample classification (Figure S3B–D) and allowing the selection of significant variables (Figure S4) which were further semi-quantitatively evaluated to biologically describe the changes in the tomato metabolome (Table S2). The preliminary results indicated that there were no significant differences when the plants were treated with the biostimulant at different application areas and concentrations (Figure S4).

Furthermore, based on pathway over-representation of the differentially abundant metabolites (in tomato leaves), 11 biological pathways were the most statistically enriched, i.e., uniquely altered by the application of the biostimulant and/or salt stress conditions. These include the biosynthesis of secondary metabolites-, linoleic acid metabolism-, and flavonoid biosynthesis pathways (Figure 2C and Table S3). These biological pathways are crucial in conference of salt stress, with roles in the synthesis of stress-responsive metabolites responsible for osmoregulation and antioxidant mechanisms (described in sections below).

**Figure 3:**
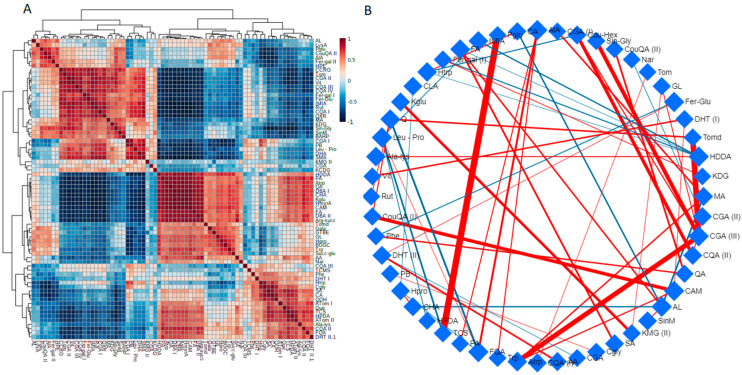
Correlation analyses among detected metabolites. (**A**) The correlation matric was obtained using the Spearman’s correlation coefficient between each pair of metabolites, the colour scheme corresponds to the colour bar, showing positive (red) and negative (blue) correlations. (**B**) Debiased sparse partial correlation (DSPC) network shows connectivity between the top 20% correlated metabolites based on *p*-value, with the cut off at 0.0005. The nodes represent metabolites, the edges represent positive (red) and negative (blue) correlations between metabolites. Edge thickness corresponds to the magnitude of the correlation coefficients. Metabolite abbreviations are provided in Table S2.

To conduct a correlation analysis for visualisation of the overall correlations between the different features to identify particular patterns under the different treatments, Spearman’s algorithm and the debiased sparse partial correlation (DSPC) algorithm were then used (Figure 3) [37]. In nature, the relationship between two metabolites can be highly complex and diverse, for instance, correlation between two metabolites could be based on their highly correlated concentrations, their participation in the same metabolic pathway, or due to the direct connection through a biochemical reaction [38,39]. However, it should be noted that two metabolites could be poorly correlated even if they are neighbours in a metabolic pathway because the modification in enzymes that control them can affect their levels in different directions, whilst apparently distant metabolites are correlated [40].

In this study, metabolite-metabolite correlations were presented in a colour-coded matrix, showing linear correlation patterns of the highest positive degree between amino acids and organic acids (red) and the lowest negative between phenolic compounds and organic acids (Figure 3A). As discussed in subsequent sections, a strong correlation between the amino acid biosynthesis pathways and the organic acid/TCA cycle in response to salt stress has been established. For better visual inspection, the DSPC network where nodes in the network are connected based on the similarity between metabolites and with edges representing the correlations, was constructed. The correlation network only shows the top 20% correlations based on their *p*-value rankings, with a cut off at 0.0005. DSPC graphical models allow discovering weighted connectivity between large numbers of metabolites, where several associations of metabolites can be discovered. On the network, very strong correlations were discovered between the flavonoid quercetin and vitexin, rutin and coumaroyl derivatives, and phenolic acids feruloyl-, coumaroyl- and sinapoyl derivatives (Figure 3B). This correlation could be based on the fact that synthesis of phenolic acids and flavonoids stem from the same metabolic pathway, the phenylpropanoid pathway (discussed in the following sections).

In general, correlation analysis can be used as proxy to describe a given physiological state of the system of interest because the correlation matrix and networks can change with the steady-state concentrations of metabolites [40,41]. Hence, it is within reason to assume that differences in the biological processes are reflected in the characteristics or patterns of the computed correlation models. Although metabolite-metabolite correlation analysis may not grasp the complete mechanisms underlying metabolic changes, it is a valuable tool for exploration of metabolomics data that permits a direct analysis of mechanistic changes in metabolism of the system under different physiological states [42].

**Figure 4:**
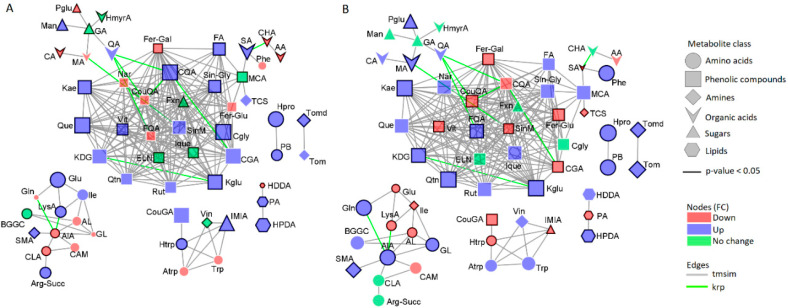
Metabolic networks displaying interconnections between chemically related metabolites in tomato plants and their relative responses to salt stress (**A**) and biostimulant treatment (**B**). The downregulation, upregulation, and no change in metabolites under different treatments are represented by red, blue and green nodes, respectively. The black borderline on the nodes indicates statistical significance (*p*-value < 0.05). Metabolite classes are represented as node shapes, as per key indicated. Interconnections between metabolites are represented by grey and green edges for chemical (tmsim) and KEGG reaction pair (krp), respectively.

In metabolomics studies, correlation analysis provides a powerful view to understand biological systems where not only individual metabolites are connected, but also their interconnections, hence providing a more comprehensive understanding of the metabolome of the system in question [39]. The global correlation networks of differentially abundant metabolites revealed two main constellations of phenolic compounds (square nodes) and amino acids (circular nodes) interconnections, based mainly on their chemical relatedness (grey edges). The metabolic networks also revealed biochemical relationships (green edges) within several phenolic compounds (naringin—coumaroyl/feruloyl/caffeoyl conjugates) and between different metabolite groups such as organic acids and phenolics (malic acid—naringin—feruloyl quinic acid) (Figure 4).

Although some amino acids including alanine, leucine, glutamine and methionine appear to have been downregulated (red) in response to salt stress (Figure 4A), others such as isoleucine, lysine and glutamate were upregulated (blue). Si-biostimulant treatment induced an overall increase in amino acid levels, while phenolic compounds were generally upregulated under both salt-induced duress and biostimulant treatment (Figure 4B). However, some organic acids and sugars including hydromystic acid and glucarate (respectively) depicted no change (green) under both salt stress and biostimulant treatment of stressed plants, suggesting a limited role in plant response to salt stress. The observed metabolic alterations in response to salt stress and biostimulant treatment are discussed in detail in subsequent sections.

The computed network-based correlation analysis provides a complementary tool to the statistical and multivariate analysis of metabolomics datasets for identification of metabolite alterations in different physiological states [39,43]. As such, this method is essential in generating hypotheses about the regulation of measured changes in metabolite profiles under different treatment conditions. Thus, correlation networks provide insight in the changes of the measured chemical space, thereby offering a further dimension to the understanding of the regulation of key metabolites in response to stressful conditions.

### 2.2. Primary Metabolism: Roles in Si-Induced Amelioration of Salt Stress Responses

The application of Si-based biostimulants on tomato plants under salt stress conditions induced shifts in primary metabolism, reflected through the reorganisation of several pathways involving fatty acids-, organic acids-, sugar- and amino acids metabolism (Table S3; Figure 2C). Primary metabolism defines the highly conserved physiological and biochemical reactions which are directly associated with normal plant growth, reproduction and energy production amongst many [44,45]. However, studies have also implicated the relevance of primary metabolism in regulation of plant defences against several stresses. It has been suggested that primary metabolism plays essential roles during responses to abiotic stress conditions, for instance, to provide energy, chemical building blocks, and as precursors of secondary metabolism [46,47]. In addition, primary metabolism has been proposed to modulate signalling waves which propagate the stress stimuli throughout the plant, leading to specialised plant defence responses [48].

#### 2.2.1. Metabolic Reprogramming of Sugars and Organic Acids in Si Treated Salt Stressed Tomato Plants

**Figure 5:**
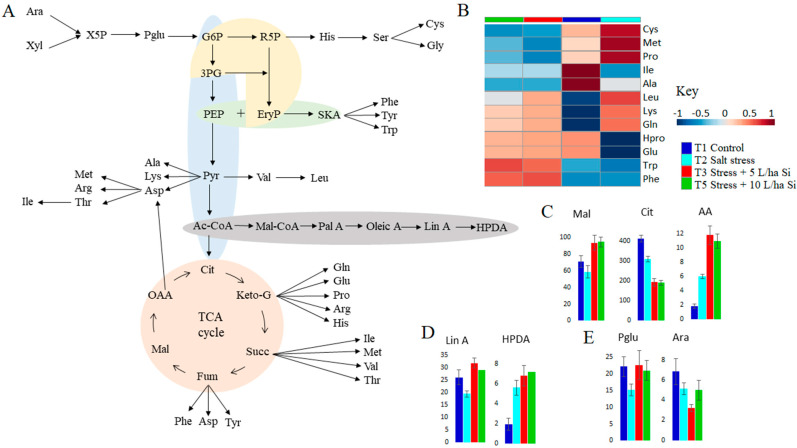
A graphical/topological representation of the intertwined pathways and relative quantification of measured primary metabolites. (**A**) The pathways incorporated include the phosphate pentose pathway (PPP) (yellow), glycolysis (blue), shikimic acid (green), organic acid/TCA cycle (brown), the fatty acid biosynthesis (grey) and several amino acid biosynthesis pathways. (**B**) Heatmap showing relative quantification of measured amino acids across non-stressed controls (T1), salt stressed (T2) and Si treated (T3 and T5) plants. Relative levels (integrated peak areas) of organic acids (**C**), fatty acids (**D**) and sugars (**E**) is depicted by bar graphs across non-stressed controls (T1), salt stressed (T2) and Si treated plants (T3 and T5), with error bars Abbreviations: Ara, arabinose; Xyl, xylose; X5P, xylose−5−phosphate; G6P, Glucose−6−phosphate; R5P, Ribose−5−phosphate; 3PG, 3−phosphoglyceric acid; EryP, Erythrose 4−phosphate; PEP, Phosphoenol pyruvate; SKA, shikimate; Pyr, Pyruvate; Cit, Citrate; Keto-G, ketoglutarate; Succ, Succinate; Fum, Fumarate; Mal, Malate; OAA, Oxaloacetate, TCA, Tricarboxylic acid.

Si-biostimulant application to salinity-stressed plants evidently alter the content of different organic compounds and sugars, e.g., reprogramming of ascorbate and alderate metabolism, sugar metabolism pathways and the TCA cycle in this current study (Table S3, Figure 2C and Figure 5). Under salt stress conditions, the untreated plants exhibited a 1.5-fold decrease compared to the non-stressed plants. On the contrary, biostimulant treatment of salt-stressed plants resulted in a relative increase of phosphogluconate levels at all time points, with doubled amounts on the first day post Si application at both 5 L/ha and 10 L/ha concentrations (Figure 5E and Figure S5). The phosphogluconate pathway, also called the pentose phosphate pathway (PPP), has been reported in numerous studies to be induced or accelerated in response to different abiotic stress conditions [49,50]. In plant cells, the PPP is considered the major metabolic defence mechanism against oxidants as it is the main source of NADPH, which feeds a variety of ROS-scavenging systems [51,52]. Under salt stress conditions, the PPP is stimulated as the main metabolic pathway, following suppression of the TCA cycle. Si-based biostimulant treatment of salt-stressed plants lead to an enhanced activity of both metabolic pathways, signifying generation of energy for both catabolic and anabolic needs of plants under stressful conditions. These results agree with the studies of [49,53] on *Zea mays* and *Swertia chirayita*, respectively, where phosphogluconate and the enzymes involved in the PPP were enhanced upon Si formulation inclusion.

On the other hand, arabinose levels were relatively lower in stressed plants compared to the non-stressed and were marginally decreased post-biostimulant treatment (Figure 5E and Figure S5). Under stressful conditions, arabinose is converted to a PPP intermediate, xylulose phosphate, which serves as a precursor for downstream metabolic energy production (Figure 5A) [54]. In addition, arabinose is also used as a carbon source for production of organic acids and some amino acids including glutamate, arginine and lysine, which feed into the TCA cycle intermediate (Figure 5A). Although there are currently no studies on Si-biostimulant effects on arabinose in stressed plants, the decreased levels of arabinose in biostimulant-treated salt-stressed plants could point to Si-mitigated stress alleviation.

In addition to sugars, several studies have reported stress-induced modifications in the levels of organic acids including most commonly malic acid, citric acid and succinic acid [55,56,57]. On the contrary to the increased levels of ascorbic acid in response to salt stress, malic and citric acids were observed to decrease considerably at all time points subsequent to salt stress (Figure 5C and Figure S5). Si application (foliar and soil) resulted in a relative increase in measured organic acids levels at different developmental stages. Citric acid and malic acid feed into the TCA cycle directly and indirectly, thereby contributing to energy production (Figure 5A). Furthermore, malate is also a source of carbon dioxide in the Calvin cycle, which is enhanced for a rapid production of glucose and other carbon skeleton donors under stressful conditions [58,59]. Similar to ascorbic acid, citric acid also has a critical role in the antioxidant responses, both act by scavenging ROS resultant of plant stress. Ascorbic acid also has other roles in plant defence mechanisms, including repair of stress-induced oxidative damage of membranes, regulation of photosynthesis, hormone biosynthesis as well as regeneration of other antioxidants in cases of elongated stress [55,60,61].

Si treatment of salt-stressed plants has been found to result in accumulation of organic acids in several plant species: *Arabidopsis thaliana* [62], *Oryza sativa* [63], *Eucalyptus* [64] and canola plants [65]. In accordance with the current study, these studies suggest that Si induced accumulation of organic acids and has stress alleviation effects on salt stressed plants. The key roles of organic acids in response to environmental stresses can be credited to their close relationships with sugars, fatty acids and amino acids, as depicted by the summarised network of amino acid biosynthesis pathways, sugar metabolism pathways (PPP and glycolysis) and the organic (citric) acid pathway in Figure 5A. Being at the centre of energy production in plants, the organic acid pathway serves as the “middleman” in the reversible pathways for degradation of amino acids and fatty acids to supply energy for the intensive energy-utilising defence metabolic reactions. According to the findings of this study, and as supported by numerous studies [66,67,68], it can be asserted that an upregulation of pathways pertaining to accumulation of sugars and organic acids is but one of the mechanisms fundamental to Si-induced salt stress alleviation in affected plants. Hence, the stress mitigation effect of Si-biostimulant can be attributed to the reconfiguration in the content of sugars and fatty acids, which lead to events including energy supply for stress-responsive cellular processes, stabilisation and repair of photosynthetic equipment resultant of salt stress and relief of oxidative stress by ROS scavenging, thus extenuating salt stress effects and improving tolerance in stressed plants.

#### 2.2.2. Si Induced Modification of Fatty Acid Metabolism in Salt Stressed Tomato Plants

Hexadecadienoic acid (HDDA) and hydroperoxyoctadecadienoic acid (HPDA) levels were altered in response to both salt stress and Si application. Salt treatment resulted in a timely decrease of HDDA levels in salt-stressed plants compared to the non-stressed plants, and the opposite was observed in HPDA levels, which were relatively increased upon longer exposure to salt stress (Figure 5D and Figure S4). Application of 5 L/ha and 10 L/ha Si both resulted in an increase of both HDDA and HPDA levels in salt-stressed tomato plants, with the most prominent upregulation of HDDA (2-fold) on the third day post-soil-applied Si treatment (Figure 5D and Figure S5). Changes in the profile of fatty acids are considered vital in stress tolerance due to the multiple defence-related roles attributed to this metabolite class. In the reaction catalysed by acetyl-CoA synthase and fatty acid synthases, citrate is converted to acetyl-CoA which, in turn, is converted to malonyl-CoA, an intermediate that is elongated into HDDA, also known as palmitic acid (Figure 5A) [69,70].

Additionally, linoleic acid and its derivatives, including HPDA, trigger lipid-dependent signalling cascades which mediate expression of defence-related genes and activate plant adaptation mechanisms [71]. These fatty acids are the main unsaturated lipids involved in stress response, specifically activating MAPKs and leading to subsequent activation of stress-responsive transcription factors [13,72]. Furthermore, HPDA can also be converted to an essential stress-responsive hormone undetected in the current study, JA [8,73,74]. Thus, HDDA, HPDA and related fatty acids regulate membrane fluidity, maintaining a state suitable for optimised integral protein function, substance exchange, and signal transduction during stress. Based on our results, supported by the literature [62,71,75], it can be postulated that one of the mechanisms by which the Si-based biostimulant alleviates salinity stress is via increasing the fatty acids content in plants. This modulation of the fatty acid content of the plant cells can lead to different molecular and physiological events, such as maintaining membrane integrity, regulating stress signalling as well providing carbon skeleton for energy production, thereby mitigating salt toxicity and improving salt tolerance in plants.

#### 2.2.3. Reconfiguration of Amino Acid Metabolism in Response to Salt Stress and Si Treatment

**Figure 6:**
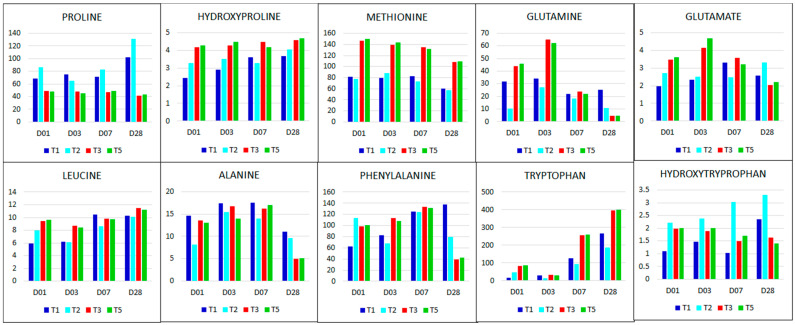
Differential changes in amino acid levels across all treatments at all time points. The column graphs were constructed using integrated peak areas (IPA) of amino acids in non-stress—(T1: dark blue), salt stress—(T2: light blue), and biostimulant-treated salt-stressed (T3: red and T5: green) conditions across all time points (days 1, 3, 7 and 28).

In the current study, the free amino acid pool was amplified post-Si treatment of the salt stressed plants, suggesting these molecules have critical roles in salt stress alleviation. This increase in amino acid content is credited to Si-induced increases in hydroxyproline, methionine, glutamine, glutamate, leucine, alanine, isoleucine, phenylalanine and tryptophan at different time points (Figure 5B and Figure 6). On the contrary, proline betaine and isoleucine levels were significantly reduced upon Si application, most notably on late stages of plant growth (day 7 and 28 post-biostimulant treatment) (Figure 6). These findings are in accordance with several studies in which amino acids accumulate significantly in salt stress conditions, and even more so upon biostimulant treatment of the stressed plants [76,77,78]. Thus, it can be postulated that several amino acids are involved in plant responses to salt stress.

Changes in the abovementioned amino acids reflect alterations of several biological pathways involved in salt stress responses, most notably the alanine, aspartate and glutamate metabolism (H), phenylalanine, tyrosine and tryptophan biosynthesis (I), arginine and proline biosynthesis (J), and the TCA cycle (M) (Table S3, Figure 2C). Rather than a linear series of reactions, amino acid biosynthesis is evidently a complex network of reactions reliant on alternative pathways. Although amino acids have primary roles as protein synthesis precursors and in donation of their carbon skeletons to TCA intermediates, most have been suggested to also have vital functions in stress alleviation mechanisms [66,79]. As displayed by the diagrammatic network on Figure 5A, the metabolism of amino acids is heavily intertwined with those of other metabolite classes, most prominently that of organic acids where amino acids reversibly feed into the TCA cycle.

**Figure 7:**
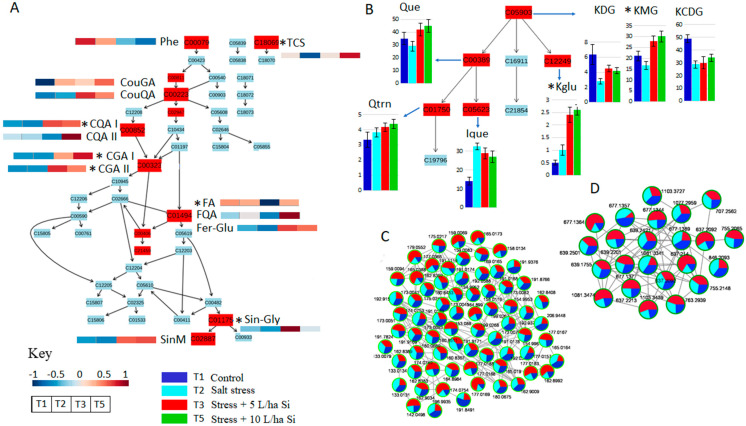
Secondary metabolic pathways and differential changes in corresponding phenolic compounds and their conjugates in non-stressed (T1: dark blue), salt-stressed (T2: light blue), and biostimulant treated salt-stressed (T3: red and T5: green) samples. The metabolite hits in the phenylpropanoid pathway (**A**) and the flavonoid biosynthesis pathway (**B**) are highlighted and matched to their corresponding graphical heatmaps representing relative amounts of each metabolite in different treatments. The asterisk (*) on metabolite titles indicate statistically significant differences (*t*-test: *p*-value < 0.05) in metabolite levels. (**C**) and (**D**), illustrate treatment-based ion abundance in molecular network clusters of cinnamic acids, flavonoids, and their derivatives, respectively.

In response to abiotic stresses, amino acids are known to act as osmolytes and precursors for synthesis of other stress-responsive molecules. While it is known for its antioxidant capabilities, proline is also classified as one of the major nitrogenous osmolyte responsible for osmoregulation under osmotic stress conditions [8,80,81]. Moreover, proline plays numerous crucial roles in plant defences, including gene reprogramming for overexpression of defence-related genes, in growth development, root growth for enhanced mineral and water absorption [8,82]. Apart from feeding into the TCA cycle intermediates (Figure 5A), leucine, alanine, and isoleucine have other roles in the physiology and metabolism of stressed plants, functioning both directly and indirectly as defence molecules which allows plants to withstand stress [83,84]. Other amino acids with essential roles in stress response are tryptophan and phenylalanine, both are produced from the shikimate pathway, which plays a fundamental role not only in plant development and reproduction, but also in plant defence mechanisms [85,86]. Tryptophan is a precursor in the biosynthesis of stress defence molecules, while phenylalanine has a major role as a precursor for the synthesis of phenolics in the phenylpropanoate pathway (Figure 7A). Hence, biostimulant-induced accumulation of tryptophan and phenylalanine in Si-formulation treated plants serves as an indicator for the upregulation of the shikimate—and phenylpropanoate pathway, suggesting stress relief [87,88,89].

These findings hence suggest that Si nutrition leads to the upregulation of several pathways including those of amino acid synthesis, and as evidenced in above subsections, that of organic acids in the TCA cycle. Such Si-based biostimulant-induced accumulation of amino acids promotes cellular osmotic adjustments, protection of photosynthesis units, and synthesis of defence-related proteins such as antioxidative enzymes and transport proteins, thus mitigating protective mechanisms and enhancing tolerance to salt stress. Moreover, with their metabolism branching out into the shikimate and phenylpropanoid pathways, amino acids (i.e., phenylalanine, tyrosine, tryptophan) form a link between primary metabolism focused mostly on plant growth, and secondary metabolism encompassing plant-environment interactions and defence-related mechanisms.

### 2.3. Si-Based Biostimulant Altered Secondary Metabolism towards the Salt-Stress Alleviation in Tomato Plants

Several studies have suggested an overlap between the primary and secondary (specialised) metabolism in response to environmental stresses [90,91,92]. A number of complex and intertwined specialised adaptive mechanisms have been reported in response to salinity stress, mainly to reduce oxidative damage caused by the stress-induced accumulation of ROS through synthesis of several compounds (phenolic acids, flavonoids, and anthocyanins) with antioxidant properties [10,93]. Hence in the current study, predictive ML-based models were built to evaluate the diversity of the specialised metabolites, revealing the most impacted phenylpropanoid—(A), secondary metabolites biosynthesis—(E), flavonoid biosynthesis—(B), and flavone and flavanol biosynthesis (D) pathways post Si-based biostimulant treatment of salt-stressed plants (Table S3, Figure 2B and Figure 7).

#### 2.3.1. Reconfiguration of Phenolics Metabolism in Response to Salt Stress

An overall increase in the content of total phenolics (phenolic acids and flavonoids) including caffeic-, coumaric-, sinapoyl- and ferulic acids and their conjugates, as well as quercetin and kaempferol conjugates was observed in response to salt treatment (Figure 7 and Figure S6). Interestingly, accumulation of these phenolics was observed on the early days of stress, with subsequent declines upon long exposure to the salt stress as observed on days seven and twenty-eight of harvest (Figure S6). This decline was observed with some phenolic acids including caffeoyl-glucaric acid isomers (CGA I and CGA II), feruloyl-galactarate isomers (Fer-Gal I and Fer-Gal II), sinapoyl-malate (SinM), and flavonoids such as kaempferol diglucosyl-glucoside (KDG), kaempferol coumaroyl-diglucosyl glucoside (KCDG), isoquercetin (Ique) and vitexin (Vit). Application of the Si biostimulant to the salt-stressed tomato plants had contrasting effects, leading to significant reductions in the content of phenolic compounds such as CGA isomers, SinM, coumaroyl-glucaric acid (CouGA), sinapoyl-glycoside (Sin-gly), caffeoyl-quinic acid isomers (CQA I and CQA IV) (Figure 7 and Figure S6). In addition, biostimulant treatment of salt-stressed plants also had variable effects on levels of flavonoids, entailing considerable declines in the levels of kaempferol glucoside (Kglu) and Ique, respectively, post biostimulant treatment (Figure 7 and Figure S6). A general decrease in flavonoid levels post biostimulant application was also observed with KDG, Vit, KCDG, and rutin (Rut).

Variable effects of biostimulant treatments on the secondary metabolism pathways involving production of phenolic compounds suggest involvement of these compounds in salt stress response mechanisms. Some of the biological pathways significantly modified include flavonoid biosynthesis and the phenylpropanoid pathways (Table S3). Phenolics are known to have remarkable antioxidant capabilities. The chemical structures of phenolic compounds are predictive of their antioxidant potentials, which circulate around radical scavenging, hydrogen- or electron- donation, and metal chelating capabilities [94,95]. In response to salt stress, phenolics act as direct ROS scavengers by donating a hydrogen atom to neutralise free radicals, as well as through a single-electron transfer. In this regard, the total number of hydroxyl groups in the phenolic compound structure influences the mechanism of antioxidant capability [89,96]. In addition, phenolic compounds also exhibit antioxidant properties by chelation of metal ions such as iron and copper, which react with hydrogen peroxide to produce a hydroxyl radicals that are known to cause DNA mutation [97,98]. Furthermore, phenolic antioxidant mechanisms include inhibition of pro-oxidant enzymes such as nitric oxide synthase (NOS), xanthine oxidoreductase (XOR) and lipoxygenase, which are responsible for stress-enhanced ROS generation [52,92,99]. Additionally, some flavonoids have been reported to show interaction with cellular defence systems through the antioxidant-responsive element (ARE), thereby stimulating antioxidant response genes including those that code for the antioxidant enzymes NADPH-quinone oxidoreductase and glutathione S-transferase [100,101].

The synthesis of phenolic compounds has been observed to be up- or downregulated in response to diverse abiotic stresses, thus leading to the increased (short-term stress) or decreased (long-term stress) total content of phenolics [11,102]. The decrease in total content upon long-term exposure to stress indicates the plant’s susceptibility to the stress, ultimately leading to cell death. In addition, reports have shown that Si treatment results in short-term accumulation of phenolics followed by a subsequent decline, signalling Si formulation-induced mitigation of salt stress effect, and hence stress alleviation [75,103]. In line with our findings, studies have also reported a Si-mitigated decline in the content of phenolics in salt-stressed apples and *Jatropha integerrima*, respectively [104,105]. In conclusion, application of Si-based formulations on salt-stressed plants confers higher tolerance thereof via accumulation of phenolics which, in turn, activate defence-related proteins, repair and regulate photosynthetic apparatus, scavenge ROS, inhibit ROS-producing enzymes as well as activate antioxidant enzymes.

#### 2.3.2. Terpenes and Polyamine Metabolism in Response to Si Treatment of Salt Stressed Plants

**Figure 8:**
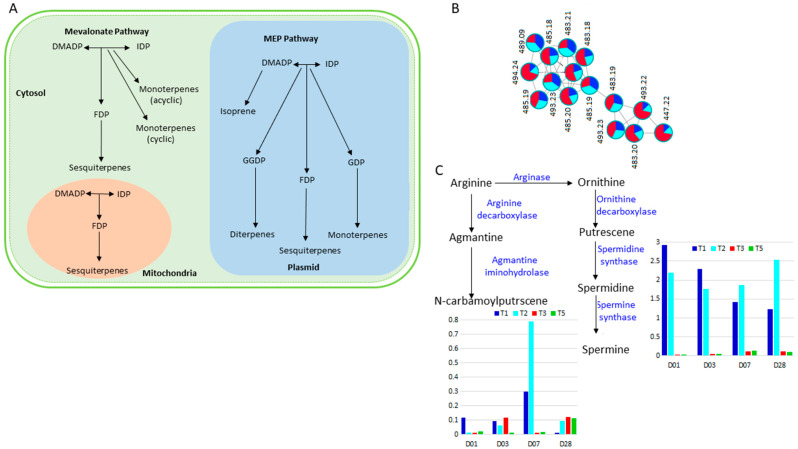
Changes in metabolism of terpenes and polyamines in response to salt stress and Si-biostimulant treatment. (**A**) A simplified representation of two main terpenes’ biosynthesis pathways: mevalonate pathway, which takes place in the cytosol (green) and the mitochondria (brown), and the 2-C-methyl-D-erythritol-4-phosphate (MEP) pathway which occurs in the plasmid (blue). (**B**) The relative abundance of the measured terpenes across non-stressed controls (dark blue), salt stressed (light blue) and Si treated plants (red) is presented as pie charts of the molecular network cluster of terpenes and derivatives (see Figure 1). (**C**) An outline of polyamine synthesis pathways and corresponding graphs showing levels of spermidine and spermine across stressed controls (T1), salt stressed (T2) and Si treated plants (T3 and T5) at all time points. Abbreviations: DMADP, dimethylallyl diphosphate; MEP pathway, 2-C-methyl-D-erythritol 4-phosphate pathway; IDP, isopentenyl diphosphate; FDP, farnesyl diphosphate; GDP, geranyl diphosphate; GGDP, geranylgeranyl diphosphate.

Along with phenolic acids and flavonoids, polyamines, terpenes and their modified constituents-terpenoids, are also secondary metabolites with critical roles in plant responses to abiotic stresses, including salinity stress. In this study, the generated molecular network (see Figure 1) revealed a cluster of terpenes and derivatives in which the quantitative pie charts showed an overall increase in salt stressed tomato plants, and greater abundance post Si treatment of the salt stressed plants (Figure 8B). On the contrary, the polyamine (tricoumaroyl spermidine and spermine) content considerably declined over time upon Si treatment of the salt stressed plants (Figure 8C).

In tomato, terpenes are found in large quantities in leaves, stems and young fruits, where the monoterpenes, diterpenes and sesquiterpenes are mainly synthesised from isopentenyl diphosphate (IDP) and dimethylallyl diphosphate (DMADP) in two pathways, the mevalonate pathway that takes place in the cytosol and mitochondria, and the 2-C-methyl-D-erythritol-4-phosphate (MEP) pathway which occurs in the plasmid (Figure 8A) [106,107]. Because of their structure entailing conjugated polyenes, these molecules have the ability to deactivate singlet oxygen and several free radicals produced in large amounts during stress duress. Terpenes and terpenoids act as hydroxyl radical scavengers, protecting the plant against stress-induced oxidative damage [108]. Furthermore, these molecules have been reported to be directly and indirectly involved in membrane stabilisation, counteracting the damaging effects of stress-induced ROS [107,109,110]. The fluctuant levels of terpenes and terpenoids are therefore indicative of the critical function played by this class of secondary metabolites in stress adaptation.

Polyamines are synthesised in the polyamine biosynthesis pathways, where the amino acids arginine and ornithine form putrescene, a precursor to the synthesis of other polyamines [111,112] (Figure 8C). Spermidine and spermine are produced from putrescene and aminopropyl residues, which are typically provided by methionine. In stressful conditions, polyamines function in concert with proline as the main osmolytes under stressful conditions [84,113]. Through adjustment of the osmotic balance, polyamines function towards alleviation of the salt-induced osmotic stress. Spermidine synchronises an array of biological processes including calcium influx, which plays a functional role in salt stress signalling [73,113]. Spermine and spermidine have also been reported to electrostatically bind with negatively charged biomacromolecules such as nucleic acids, acidic proteins and membrane phospholipids, allowing them to regulate expression of defence-responsive genes, enzyme activity as well as stabilise the membranes for optimised functioning of channel proteins and exchange of substances [84,114]. In addition, polyamines enhance photosynthetic activity and the deactivation of ROS, hence reducing the inhibitory effects of salt stress [115]. Spermidine levels have also been reported to be elevated under numerous abiotic stress conditions with supporting evidence of its role in regulation of expression genes that are involved in osmotic stress tolerance and as signalling molecules [84,114]. Thus, Si-based biostimulant treatment of salt-stressed tomato plants ameliorates the stress effects via several polyamine- and terpene mediated mechanisms including protection of macromolecules, osmotic adjustments and detoxification of ROS.

**Figure 9:**
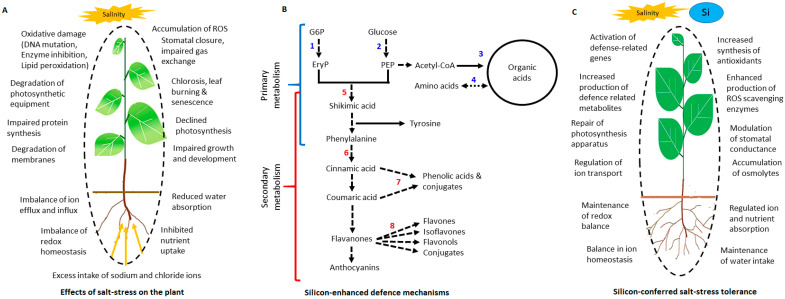
A postulated overview of Si-mitigated alleviation of salt stress effects on tomato plants via modulation of the primary and secondary metabolic pathways. (**A**) Scheme portraying the detrimental effects of salt stress on a plant. Excess accumulation of Na^+^ and Cl^−^ ions in the soil, hence an increased absorption causes an imbalance in the uptake of other ions, water and nutrients. Subsequent ionic and osmotic stresses enhance synthesis of ROS, resultant in oxidative damage of macromolecules and photosynthetic equipment. (**B**) Si-based biostimulant treatment of the salt-stressed plants leads to modifications in the slightly overlapping primary (red) and secondary (blue) metabolic pathways for a coherent defence response. Upregulation of these pathways result in accumulation of osmolytes, enhanced synthesis of enzymatic and non-enzymatic antioxidants, ROS detoxification and repair of the salt-stress induced oxidative damage. Coherent activities of the Si-enhanced defence responses ultimately lead to conferred salt stress tolerance in plants (**C**). Primary metabolic pathways (blue): glycolysis (1), pentose phosphate pathway (2), tricarboxylic acid cycle (3), amino acid biosynthesis pathways (4) and the secondary metabolic pathways (red): including shikimate pathway (5), phenylpropanoid pathway (6, 7) and flavone and flavonol biosynthesis pathways (8). Abbreviations: G6P, Glucose-6-phosphate; EryP, Erythrose phosphate; PEP, Phosphoenol pyruvate.

Plants are frequently exposed to the adversity of abiotic stresses which have hostile effects on plant growth and development. Evolutionarily, plants have adapted several stress response mechanisms based on the intrinsic metabolic capabilities, to cope with the rapid fluctuations. Such protective mechanisms induce cellular metabolic reprogramming to facilitate the bio-physio-chemical pathways in response to the external environment. Thus, our findings evidenced that the key mechanisms induced by the Si-biostimulant towards stress alleviation encompassed the primary metabolic reactions such as glycolysis, TCA cycle, fatty acid- and amino acid biosynthesis pathways (Figure 9). In addition, the more specialised secondary metabolism was also impacted by the biostimulant application through activation of stress-responsive pathways including the phenylpropanoid pathway, flavonoid biosynthesis pathways and other secondary metabolites biosynthesis pathways (Figure 9).

The postulated model (Figure 9) therefore suggests that Si-based biostimulant confers salt stress tolerance through reconfiguration of the plant’s metabolome to synthesise stress-responsive molecules. Si-induced accumulation of several amino acids, organic acids, fatty acids and sugars have been suggested to confer salt stress as osmoprotectants in the first line of response in plants during salinity, hence alleviating the resultant osmotic stress. Furthermore, these metabolites feed into the TCA cycle for biosynthesis of various osmotic regulators and an uninterrupted supply of energy needed for the sequestration of toxic Na^+^. In addition, Si nutrition resulted in increased production of secondary metabolites comprising phenolic acids, flavonoids, polyamines and terpenes which are mainly important antioxidants, functioning coherently with the primary metabolites to scavenge the salt stress-resultant ROS species, hence improving the oxidative damage. These findings were reflected by the agronomic and phenotypic data, some of which reported in this study (Figure S7); where Si treatment of the salt stressed plants translated to an increase in the plant height, dry root biomass and the number of tomatoes fruits. In addition, increased content, and uptake of several nutrients including Ca^2+^, Cu^2+^, Fe^2+^, Na^+^, S^2−^, and Mn^2+^ was measured post Si treatment of the stressed plants. Ultimately, the stress and biostimulant-induced metabolic reprogramming leads to several physiological mechanisms that counteract the salt stress effects including activation of defence-related genes, repair of the stress-induced damage on the photosynthesis apparatus, stomatal closure, enhanced rate of ROS scavenging and regulation of ion and nutrient absorption (Figure 9). As such, Si-based biostimulant treatment of salt stressed plants has been shown in this study, to alleviate salt stress effects and promote tolerance at the developmental stages of tomato plants.

## 3. Materials and Methods

All reagents used from the pre-analytical to data acquisition stages were purchased from international suppliers in pure or analytical grade quality. Organic solvents used in metabolite extraction and data acquisition (acetonitrile and methanol) were of LC-MS grade, from Romil, SPS, (Cambridge, UK). Leucine encephalin and formic acid were acquired from Sigma Aldrich (Munich, Germany), while the water was purified using an in-house Milli-Q Gradient A10 system (Siemens, Fahrenburg, Germany).

### 3.1. Plant Cultivation and Treatments

Hybrid tomatoes (*Solanum lycopersium*) STAR 9009 were planted in 10 L-pots filled with coir (20 seeds per pot) and positioned randomly (on rotating tables) in a greenhouse located at Omnia Group facilities in Sasolburg, Free State, South Africa. The study was designed as a three-factorial experiment laid out in a split-plot on rotating tables. At emergence, the plants were thinned to 5 plants per pot based on uniformity and the health of the plants. Each pot was considered as a biological replicate and contained five plants at the harvesting time. The study comprised controls and treated groups, all referred to as treatments (T) (Table 1). Control groups consisted of T1, T2 (no biostimulant application); and treated groups included T3–6 (salt stress and biostimulant applications). Five biological replicates (i.e., five pots) per treatment were harvested at each time point (Table 1). The tomato plants (at 4th leave stage) were exposed to salt stress in a form of sodium chloride (NaCl) 10 days prior to biostimulant treatment. The first irrigation, to prevent salt shock by the plants, was application of 25 mmol/L of NaCl. Thereafter, the concentration of the salt was doubled to 50 mmol/L for the duration of the trial.

Ten days post 50 mmol/L salt stress induction, the plants were treated with a Si-based biostimulant formulation (Omnia Group Ltd., Bryanston, South Africa) through soil and leaf (foliar) application (Table 1). The biostimulant formulation used in this study is Fortisil-K, a soluble potassium silicate product (Omnia Group Ltd., Bryanston, South Africa). Here, for semantics simplicity, the formulation will be referred to as Si-based biostimulant or simply the biostimulant or Si-formulation. The biological perturbations due to the applied treatments were monitored over a total of 4 w post-treatment (WPT).

### 3.2. Harvesting Plant Materials and Metabolite Extraction

The leaves of the tomato plants were harvested at 4 different time points: 1, 3, 7, and 28 d post-biostimulant treatment (DPT), for all treatments and biological replicates. The leaves were picked randomly and immediately immersed in liquid nitrogen to rapidly quench enzymatic activity. The leaves were then stored at −20 °C until further analysis. Metabolites were extracted from leaves from all treatments and biological replicates. Liquid nitrogen was added to the leaves (which were kept at −20 °C), followed by grinding of the frozen material to a powder form using a pestle and mortar. To avoid any chance of sample crossover, the pestle and mortar were cleaned (washed using dH_2_O and rinsed with 80% aqueous methanol) between samples.

Two grams per sample were weighed into a clean Falcon tube and 20 mL of 80% cold (aqueous) methanol (4 °C) was added (1:10 *m*/*v* ratio). The mixture was homogenised for 2 min using an Ultra-Turrax homogeniser and sonicated for 30 s with a probe sonicator (Bandelin Sonopuls, Berlin, Germany) at 55% power. The homogeniser and the probe were cleaned with 80% aqueous methanol between samples to avoid sample crossover. All homogenates were centrifuged at 5100 rpm (5525× *g*) for 15 min at 4 °C. The supernatant from all samples were each concentrated to 1 mL using the Büchi Rotavapor R-200 at 55 °C, and dried to completeness with a speed vacuum concentrator (Eppendorf, Merck, South Africa) set at 45 °C. The dried samples were re-suspended in 500 µL of 50% aqueous methanol (LC-MS grade). All sample suspensions were filtered through 0.22-µm nylon syringe filters into pre-labelled HPLC glass vials fitted with 500 µL inserts (Shimadzu, Johannesburg, South Africa). Pooled quality control (QC) samples were prepared by pipetting equal volumes from each aliquoted sample. All samples were stored at −4 °C until downstream analysis.

### 3.3. Data Acquisition Using Ultra-High-Performance Liquid Chromatography-High Definition Mass Spectrometry (UHPLC-HDMS)

Data was acquired through subjection of the aqueous-methanol extracts to UHPLC-HDMS analyses on a Waters Acquity UHPLC system coupled to a SYNAPT G1 High Definition (HD) quadrupole time-of-flight (Q-TOF) mass spectrometer (Waters Corporation, Milford, MA, USA). Chromatographic separation of the analytes in samples was done on a HSS T3 C18 reverse-phase column (150 mm × 2.1 mm × 1.8 µm) (Waters Corporation, Milford, MA, USA), thermostatted at 60 °C, with 3 replicated injections of 3 µL per sample. A gradient elution method was employed at a flow rate of 0.4 mL/min, with solution A (0.1% aqueous formic acid) and solution B (0.1% formic acid and acetonitrile—Romil Pure Chemistry, Cambridge, UK). Elution of solution B in the binary solvent commenced at 2% (*v/v*) for 0–1 min, increased to 70% from 1–14 min and further to 95% from 14–17 min. To clean up and equilibrate the column, elution of solution B was decreased to 2% in the last minutes of the run (17–20 min). An analysis of solvent blanks and QC samples was done in parallel with the tomato extracted metabolite samples.

For mass spectrometry analyses, the electrospray ionisation (ESI) source (of the SYNAPT G1 MS system) operated in both negative and positive modes. The mass analyser—time-of-flight (TOF)—was operated in V-optics. The centroid data was acquired with the scan range of 50–1200 Da, with the scan time of 0.1 s and an inter-scan delay of 0.02 s. Leucine enkephalin solution (50 pg/mL), [M + H]^+^ = 556.2766 and [M − H]^−^ = 554.2615, was continuously sampled to ensure acquisition of high mass accuracy (1–3 mDa) of analytes via MassLynxTM software automatic corrections of small deviations from the exact mass value. This standard solution was sampled every 15 s, producing an average intensity of 350 counts per scan in centroid mode. Other mass analyses parameters entailed: source temperature set at 120 °C and desolvation temperature at 450 °C, with capillary-, sampling cone-, and extraction cone voltages at 2.5 kV, 30 V and 4 V, respectively. Nitrogen gas was the nebulisation gas at a flow rate of 700 L/h. A data-independent acquisition (DIA) method, MS^E^/MS^All^, was applied, using alternating low-energy collision-induced dissociation (CID) and high-energy CID: i.e., full scan (non-fragmented, 0 eV) and five high-energy CID (10–50 eV). The low-energy CID is to obtain precursor ion mass spectra, and the high-energy CID is used to acquire product ion (fragmentation) information. The collected fragmentation data were used for downstream compound identification and structural elucidation. All data was manipulated using MassLynxTM 4.1 (SCN 704, Waters Corporation Milford, MA, USA).

Pooled quality control (QC) samples were used for assessment of the reliability and reproducibility of the data generated, as well as for corrections of the non-linear signals. A QC sample was injected after each 10 randomised sample injections to accurately monitor the deviations caused by the instrument. In addition, 6 injections of a QC sample were done at the beginning and the end of each batch to ensure equilibration of the system and reduce bias in the measurements. The blank samples (consisting of 50% methanol) were also randomly run to detect any background noise.

### 3.4. Data Mining: Data Processing, Multivariate Data Exploration and Machine Learning-Based Classification

Centroid raw data acquired from both positive and negative ESI modes was visualised and pre-processed using MassLynx XSTM 4.1 (Waters Corporation, Manchester, UK), generating a data matrix of paired retention time (Rt) and m/z variables, with their respective peak intensities. For data processing, the MarkerLynx software parameters were set to process the m/z domain of 100–1200 Da within the retention time (Rt) range of 0.9–14 min. Mass tolerance of 0.05 Da and intensity threshold counts of 100 were set for both positive and negative data, with a Rt window of 0.2 min. Prior to computation of peak intensities, MarkerLynx software was set to execute a modified Savitzky-Golay smoothing and integration, retaining only data matrices with the noise levels below 10% (as determined by MarkerLynx) for subsequent data analysis. After data extraction (by MarkerLynx), a mandatory data scrutiny was meticulously done, including assessment of the number of extracted features (˂10,000 features, as a rule of thumb), applying the 80% rule (i.e., features found in less than 20% of the analysed samples were removed) and monitoring the quality of data and stability of the analysis using QC samples. Data transformation methods—centering, scaling or transformation—were ‘exploratively’ employed to put all variables on equal footing, minimise variable redundancy and adjust for measurement errors.

The MassLynx generated data matrices were exported to SIMCA (soft independent modelling of class analogy) software, version 15 (Umetrics, Umeå, Sweden) and a web-based data analysis tool, MetaboAnalyst version 4.0 (Chong et al., 2018) for data mining and interpretation. The approach followed in this study was a chemometric approach: (i) firstly, exploration of the data using unsupervised chemometrics methods, such as principal component analysis (PCA), to reduce the dimensionality of the data, evaluate the structures and characteristics of the data (natural groupings, trends and outliers); (ii) following unsupervised modelling, and based on the insights extracted from it, supervised multivariate hybrid machine learning (ML)/classical statistical methods such as orthogonal projection to latent structures-discriminant analysis (OPLS-DA) were applied. This allowed for sample classification (in low dimensional space) as well as the identification and selection of variables (metabolite features) underlying the discrimination between groups or classes. Different metrics and tests were applied for model validation, and these include evaluation of explained and predicted variation (cumulative R2 and Q2), cross-validation analysis of variance (CV-ANOVA) and permutation testing.

Prior to computing chemometric and ML models, pre-treatment methods such as data transformation and Pareto-scaling were applied to normalise and adjust all the variables to a comparable footing. An adjusted non-linear iterative partial least square (NIPALS) algorithm was used to manage the missing values, with a correction factor of 3.0 and a default threshold of 50% [116]. A seven-fold cross-validation (CV) method was applied as a tuning procedure in model computations. This *k*-fold cross-validation is the most common method for model evaluation and selection in machine learning, where a dataset is iterated k times. In each iteration, the dataset is divided into k parts: validation and training subsets. This implies that, in the case of a 7-fold CV, for a fixed number of components, the Y values of all individuals of each subset are predicted using a sub-model computed with the 6 other subsets. Results of this k-fold cross-validation procedure are summarised by quality parameters, such as R2 and Q2 metrics in this case. R2 indicates the explained variation (i.e., goodness of fit) and Q2 refers to predicted variation (i.e., the predictive ability of the model) [117,118].

### 3.5. Molecular Networking

The raw MS/MS data was converted to ‘analysis base file’ (ABF) format using the Reifys Abf converter software (https://www.reifycs.com/AbfConverter (accessed on 6 June 2021)) then uploaded into the Mass Spectrometry-Data Independent AnaLysis (MS-DIAL) software (http://prime.psc.riken.jp/compms/msdial/main.html (accessed on 21 June 2021)). The MS-DIAL data-processing program uses the deconvolution algorithm to perform mass spectral deconvolution of data-independent acquisition (DIA) data, thus making it applicable for the extensive untargeted metabolomics analysis of both DIA and data-dependent acquisition (DDA) centroid datasets [119]. The data were processed using the following parameters: mass accuracy (MS1 and MS2 tolerance) of 0.05 Da, minimum peak height of 10 amplitude and mass slice width of 0.05 Da for peak detection, a 0.5 sigma window value and a 0 MS/MS abundance cut-off for data deconvolution; a retention time tolerance of 0.05 min was used under alignment parameter settings with one of the QC samples used as a reference file for alignment. The GNPS (Global Natural Products Social Molecular Networking) files (GnpsMgf and GnpsTable/feature quantification table were exported and uploaded into the GNPS environment (https://gnps.ucsd.edu/ (accessed on 21 June 2021)) using the WinSCP server for molecular networking.

The respective feature quantification table, MGF file and a metadata file describing the properties of the sample file (i.e., treatment, days, plant condition, Si-biostimulant concentration and stress level) were uploaded for generation of a feature-based molecular network (FBMN). The MS/MS fragmentation spectra were assembled using the MS-Cluster algorithm with a precursor ion mass tolerance of 0.05 Da and fragment ion mass tolerance of 0.05 Da. A network was generated where the lines/edges connecting the nodes were filtered to have a cosine score above 0.7 and a minimum of 4 corresponding fragment ions. The MN spectra were then searched against the spectral libraries housed in GNPS where the same parameters (i.e., cosine score > 0.7 and min-matched fragments of 4) were used for metabolite annotation. To improve the chemical structural annotations, the generated molecular network data was enhanced with MolNetEnhancer then visualised on the Cytoscape (https://cytoscape.org/ (accessed on 30 September 2021)) network visualization tool/software (version 3.8.2), where the nodes were labelled with the precursor mass (*m*/*z*) and coloured based on metabolite class. The individual nodes, representative of measured ions, were also coloured to display colour-coded pie charts illustrating relative ion abundance across different treatments. The edges were coloured based on the type of interaction: Kyoto Encyclopaedia of Genes and Genomes (KEGG) reactant repair (krp) and the Tanimoto chemical similarity (tmsim) between the metabolites. The fragmentation spectra of all the putatively annotated metabolites matched to the GNPS spectral libraries were manually validated as described in the next session.

### 3.6. Metabolite Annotation and Biological Interpretation

The chemometrically and statistically selected variables (spectral features) from data modelling were confidently annotated through a multistep workflow, and metabolites were annotated to the level 2 as classified by the Metabolomics Standard Initiative (MSI) (Sumner et al., 2007). Thus, the main steps followed for metabolite annotation in this study included: (i) computation of the molecular formula (MF) based on mass accuracy and the golden heuristic rules integrated in MarkerLynx formula generator algorithms (i.e., nitrogen rules, mass variances, isotopic fit, ring double bond equivalents (RDBE) and element number restrictions); (ii) the computed MF were manually and automatically searched against various databases such as KEGG (https://www.genome.jp/kegg/ (accessed on 18 October 2020)), Dictionary of Natural Product (DNP), ChemSpider (http://www.chemspider.com/ (accessed on 12 April 2020)) [120] and an in-house library to putatively assign compound names to the MF; (iii) structural elucidation was executed through careful evaluation of fragmentation patterns on MS1 and MSE spectra of selected metabolite candidates; (iv) structural confirmation was done by comparative assessment of in silico and experimental fragmentation information, searching against in-house spectral library and annotation details (of a metabolite under consideration) reported in the literature. Furthermore, metabolic pathway and network analysis of the annotated metabolites (and targeted compounds) was done using the MetPA (Metabolomics Pathway Analysis) module of the MetaboAnalyst bioinformatics tool suite (version 4.0) (https://www.metaboanalyst.ca/ (accessed on 17 August 2021)) [121], which allowed for the extraction of the impacted metabolic pathways, analysis thereof and visualisation. In addition, KEGG was also used for construction of web-works to enable analysis, visualisation, integration and understanding of the complex mechanisms underlying Si effects on salt-stressed tomato plants. A correlation network was constructed from MetaMapp-encoded chemical structures of all identified metabolites retrieved from the PubChem and KEGG databases using MetaMapp (http://metamapp.fiehnlab.ucdavis.edu/ (accessed on 30 August 2021)) and visualized on Cytoscape v3.8.1 for a global view of the metabolic changes across different treatments.

## 4. Conclusions

Identifying and delving into the regulatory mechanisms of biostimulants in improving plant stress tolerance will help fine-tune the most effective application rates, areas, concentrations, and biostimulant-plant specificities which may collectively yield the highest impact on stress protection. Fundamental knowledge and understanding of such mechanisms are pivotal for the development and generation of biostimulants, where synergies of microbial and/or nonmicrobial mechanisms are functionally and complementarily designed, offering the potential to overcome specific and general stresses to a spectrum of plant species. A comprehensive and systematic approach to discover the mode of action of biostimulants has been proposed—metabolomics. This multidisciplinary omics technique can be used to interrogate cellular biochemistry, providing a window to understand cellular and molecular language in the context of biostimulant-plant interactions. As such, metabolomics reveals mechanisms that define biostimulant activity in stress alleviation. Meta-analysis of the Si-biostimulant-mitigated salt stress alleviation was conducted in this study.

The predictive models derived show positive modulations in the primary and secondary mechanisms of Si-biostimulant-treated plants under salt stress conditions, including reconfigurations in the metabolite profiles of amino acids, fatty acids, organic acids, and phenolics. The analysis found that Si-biostimulant application induces accumulation of primary metabolites in such treated plants, which collectively replenish the energy for several metabolic reactions, modulate stress signalling waves that lead to specialised plant stress responses, act as osmotic and photosynthetic protectants and are precursors for the more defence-oriented secondary metabolic reactions. Moreover, Si-biostimulant is postulated to confer salt stress tolerance via accumulation of phenolics, as indicated by the timely decrease in their content which points to stress alleviation. These specialised metabolites define a huge part of secondary metabolism with defence-related roles including ROS scavenging, activation of defence-related proteins and antioxidant enzymes, and inhibition of ROS-generating enzymes. Such metabolite reprogramming spans several biostimulant-impacted primary metabolism pathways including the TCA cycle, amino acid- and fatty acid biosynthesis pathways, the PPP and secondary metabolic mechanisms such as the phenylpropanoid pathway, flavonoid biosynthesis pathway, and other secondary metabolite biosynthesis pathways.

As illuminating as the current study is, emphasis of future studies for an expanded and hypothesis testing could be extended to (i) a targeted analysis focused on the proposed mechanisms that define a nonmicrobial biostimulant activity, (ii) development of integrated omics technologies (genomics, transcriptomics, proteomics, metabolomics) research for a systems biology approach towards biostimulants and (iii) synergistic effects of microbial and/or nonmicrobial biostimulant formulations against a plethora of biotic and abiotic stresses.

## Figures and Tables

**Table 1:** Description of treatments and their corresponding Si-based biostimulant application rates.

Treatment (T)	Treatment Description	Application Rate (L/ha)
T1	Control: No salt stress application, no biostimulant application	0
T2	Control: Salt-stress application and no biostimulant application	0
T3	Salt stress application and soil applied biostimulant	5
T4	Salt stress application and foliar applied biostimulant	5
T5	Salt stress application and soil applied biostimulant	10
T6	Salt stress application and foliar applied biostimulant	10

## Data Availability

The data presented in this study are available on request from the corresponding author. The (raw) data are not publicly available due to follow-up studies.

## Supplementary Materials

The following are available online at https://www.mdpi.com/article/10.3390/metabo11120820/s1, Figure S1: Representative UHPLC-MS BPI chromatograms from the ESI negative data showing treatment-related metabolic variations, Figure S2: Unsupervised machine learning (ML)-based chemometric modelling of the ESI negative data—principal component analysis (PCA) scores plots, Figure S3: Representation of supervised machine learning (ML)-based orthogonal projection of latent structures discriminant analysis (OPLS-DA) modelling of the data, Figure S4: Identification of the statistically significant features/metabolites in different treatments discriminations using the variable importance in projections (VIP) plots, Figure S5: Summarised effects of silicon treatment of salt-stressed plants on detected primary metabolism components of organic acids (MA, AA, CA), sugars (Pglu, Ara) and fatty acids (HDDA, HPDA), Figure S6: An overview of relative phenolics and polyamine content fluctuations in different treatments, Figure S7: The effects of different treatments on the agronomic and phenotypic states of the plant samples. Table S1: Metamapp data used for generation of a network (Figure 4), Table S2: An overview of all metabolites detected and annotated from all different treatments (T1–T6) at all timeA-points_days 1, 3, 7 and 28), Table S3: The most significantly altered pathways based on impact factor >0.1, Holm adjusted *p*-value < 0.05 and FDR < 0.05. The indication letters correspond to the labelled pathways on the pathway impact plot (Figure 3B).
