# Supplementary material for: A Global Metabolic Map Defines the Effects of a Si-Based Biostimulant on Tomato Plants under Normal and Saline Conditions
Source: Metabolites. 2021 Nov 30;11(12):820. doi: 10.3390/metabo11120820 (PMC8709197; doi:10.3390/metabo11120820)
Supplement: Supplementary file 1 [file metabolites-11-00820-s001.zip › metabolites-1445829-supplementary.pdf]

SUPPLEMENTARY MATERIALS

The supplementary figures and tables are provided as additional information to support the main results reported in this study. All the experimental raw data, figures and tables that are not included in this manuscript are available from the corresponding author, Dr Fidele Tugizimana, Department of Biochemistry at the University of Johannesburg.

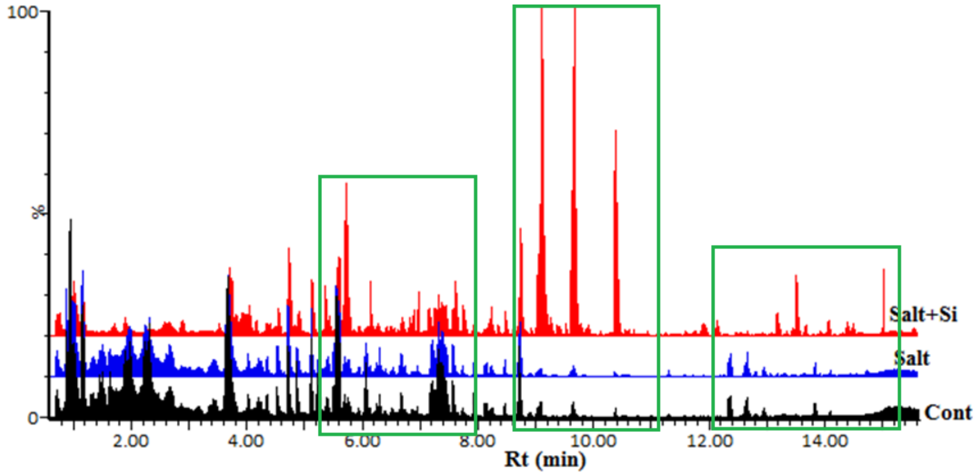

Figure S1: Representative UHPLC-MS BPI chromatograms from the ESI negative data showing treatment-related metabolic variations. The overlaid chromatograms display several ion peaks reflective of the complexity of the biological leaf extracts from non-stressed controls (black), naïve salt-stressed samples (blue), and silicon-treated salt-stressed samples (red). Visual inspection of the highlighted regions on the chromatograms shows differential peak intensities between the treatments, as well as the absence and presence of some peaks.

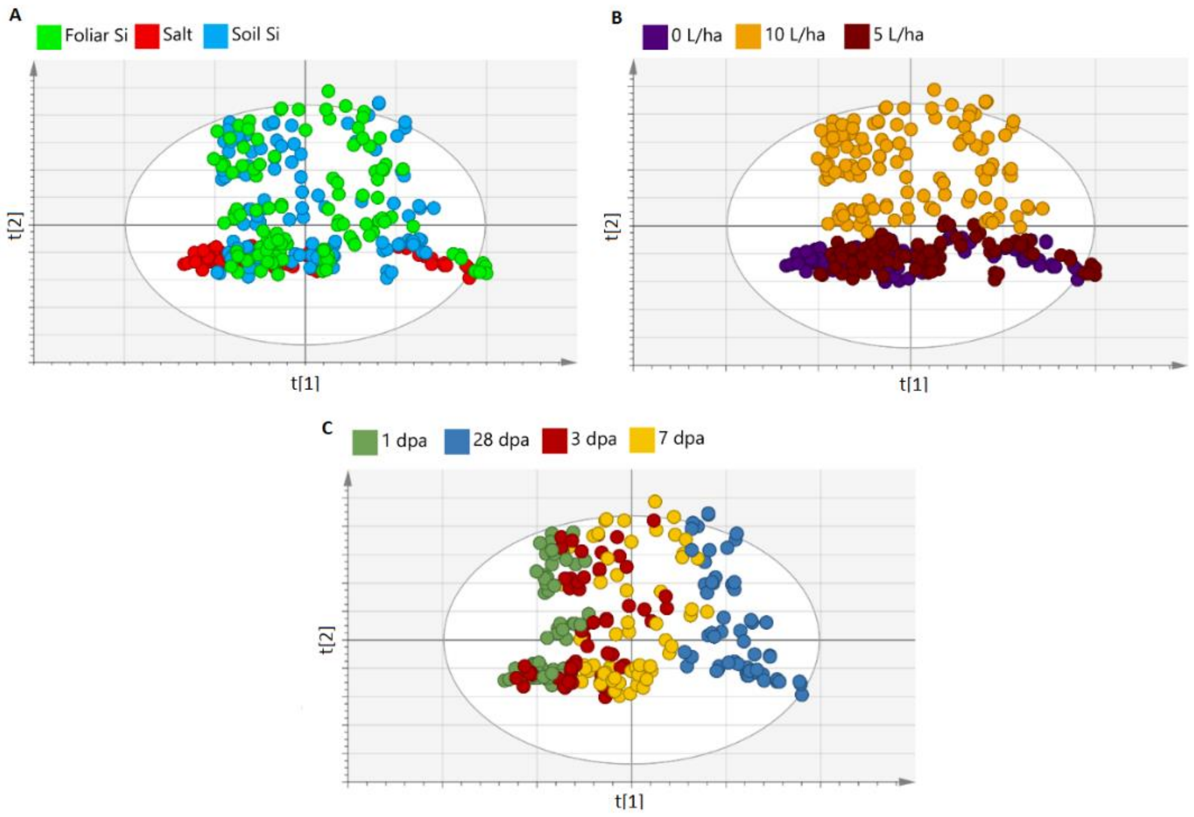

Figure S2: Unsupervised machine learning (ML)-based chemometric modelling of the ESI negative data – principal component analysis (PCA) scores plots. The PCA scores plots provide a visual overview of the multi-dimensional data, allowing identification of natural treatment-related sample groupings and patterns. The 18-component PCA model computed from the Pareto-scaled data exhibit 0.802 explained variation (R^2) and 0.653 predicted variation (Q^2), according to the 7-fold cross validation. Visual inspection reveals that silicon treatment of salt-stressed samples has an effect regardless of area of application (**A**), concentration of Si-based formulation (**B**) and a time points investigated (**C**).

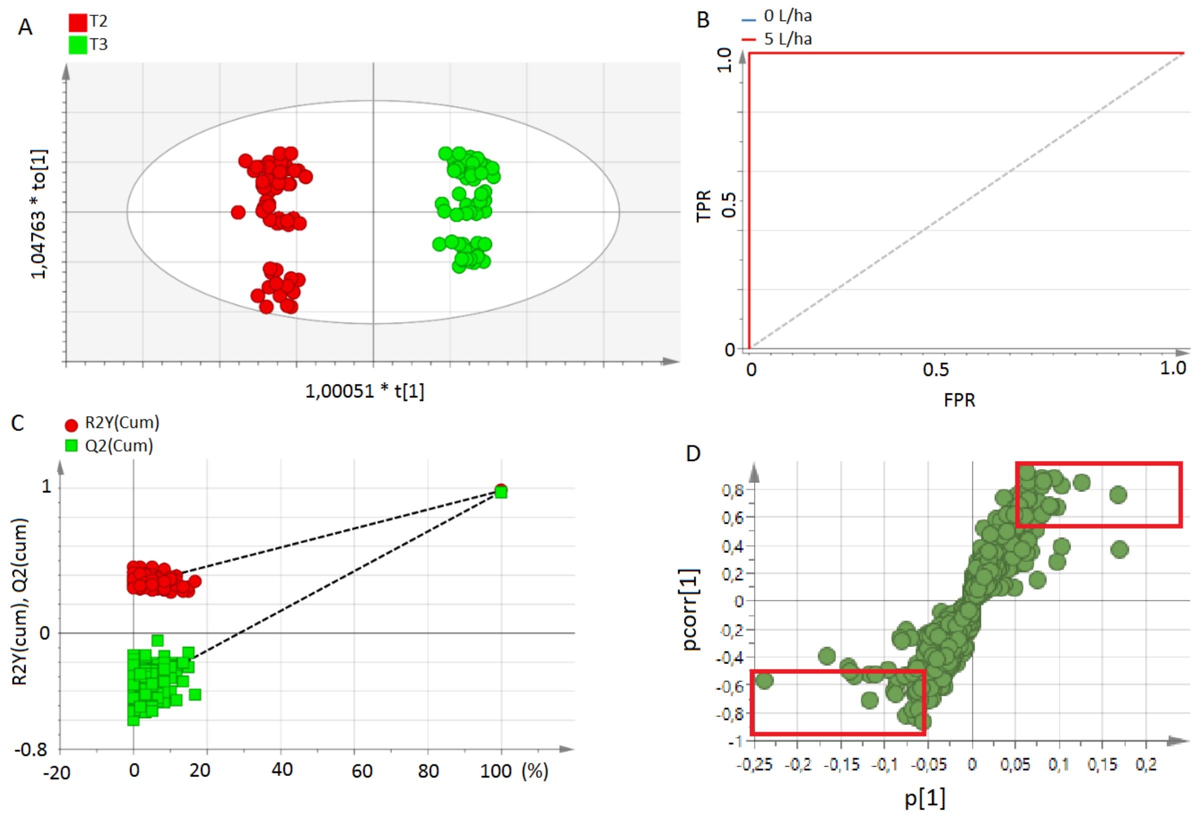

Figure S3: Representation of supervised machine learning (ML)-based orthogonal projection of latent structures discriminant analysis (OPLS-DA) modelling of the data. This supervised regression provided a visual and supervised classification of the data groups and permits extraction of statistically significant metabolites in the discrimination of naïve salt-stressed samples (T2 - red) and the biostimulant-treated salt-stressed samples (T3 - green), as presented on the OPLS-DA scores plot (**A)**. The model was validated by assessing its robustness (**B**: receiver operator characteristic (ROC) curves), predictive ability and reliability (**C**: permutation test), and its significance using cross-validated analysis of variance (CV-ANOVA) tests. The scrutinised models were found to be very good classifiers with 100% sensitivity and 100% specificity (**B**), and none of the permuted models were found to have better performance than the selected ones (**C**), with cumulative R2Y and Q2 > 0.95 (0.983 and 0.976, respectively in the presented model), and reliable statistical significance (CV-ANOVA: p-value < 0.05). The constructed OPLS-DA loadings S-plot (**D**) was used to identify statistically significant metabolites/variables with both high correlation and covariance, as indicated on the figure (red) rectangles.

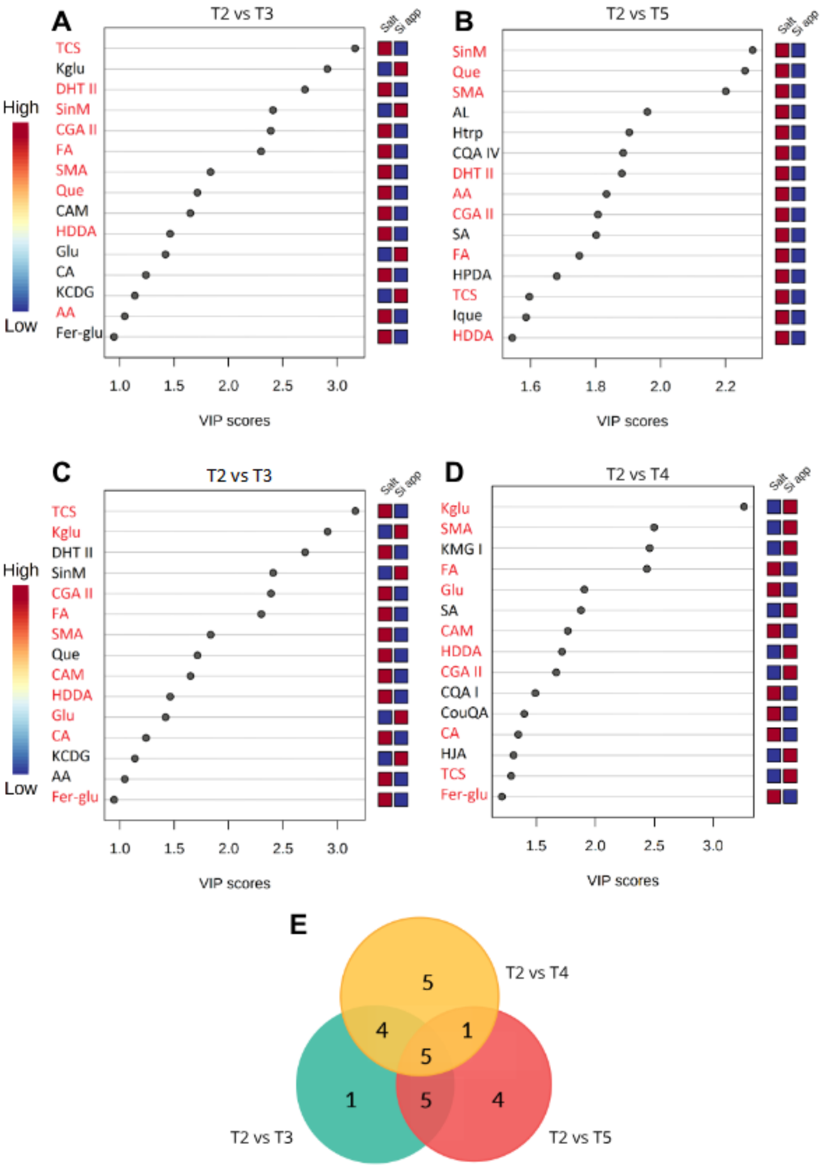

Figure S4: Identification of the statistically significant features/metabolites in different treatments discriminations using the variable importance in projections (VIP) plots. Important metabolites in the discriminations between treatments were identified using VIP computations (in accordance with the OPLD-DA S-loadings plot, the higher the VIP score (>1), the more significant the metabolite in the classification. The plots were used to investigate the effect of silicon-treatment on salt-stressed plants based on formulation concentration: **A** (5 L/ha soil applied Si-based biostimulant) vs. **B** (10 L/ha soil applied Si), and on Si-application area **C** (5 L/ha soil applied Si-based biostimulant) vs. **D** (5 L/ha foliar applied Si-based biostimulant). The identified common metabolites between the treatment comparisons were highlighted in red and presented in a Venn diagram (**E**). The 5 L/ha soil applied Si-formulation (T3) exhibits more common metabolites with both the 10 L/ha soil applied Si-based biostimulant and the 5 L/ha foliar applied Si-based biostimulant.

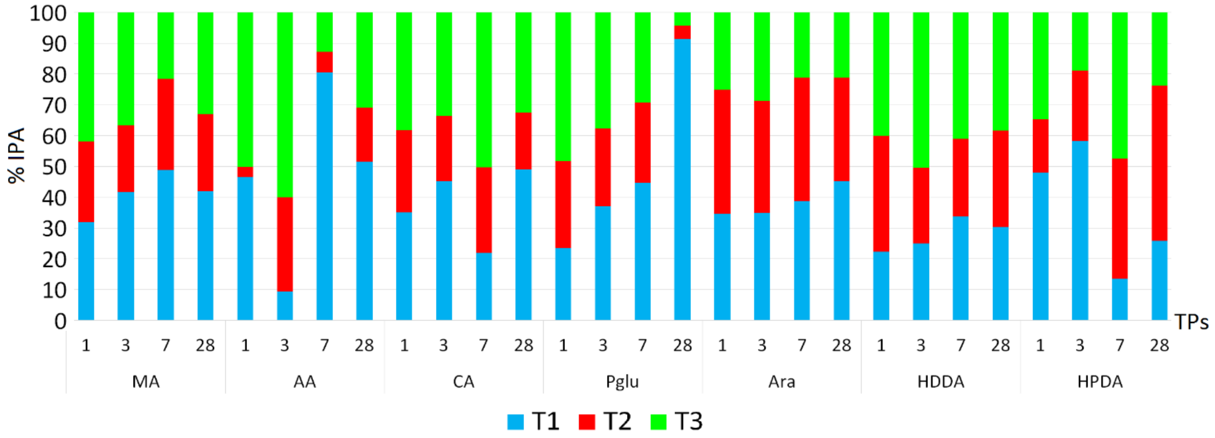

Figure S5: Summarised effects of silicon treatment of salt-stressed plants on detected primary metabolism components of organic acids (MA, AA, CA), sugars (Pglu, Ara) and fatty acids (HDDA, HPDA). The constructed stacked column graphs display relative integrated peak area percentages (% IPA) of metabolites across non-stress- (T1, blue), naïve salt stress- (T2, red) and biostimulant treated salt stress (T3, green) conditions across all time points (days 1, 3, and 28). **Abbreviations:** MA, malic acid; AA, ascorbic acid; CA, citric acid; Pglu, phosphogluconate; Ara, arabinose; HDDA, hexadecadienoic acid; HPDA, hydroperoxyoctadecadienoic acid.

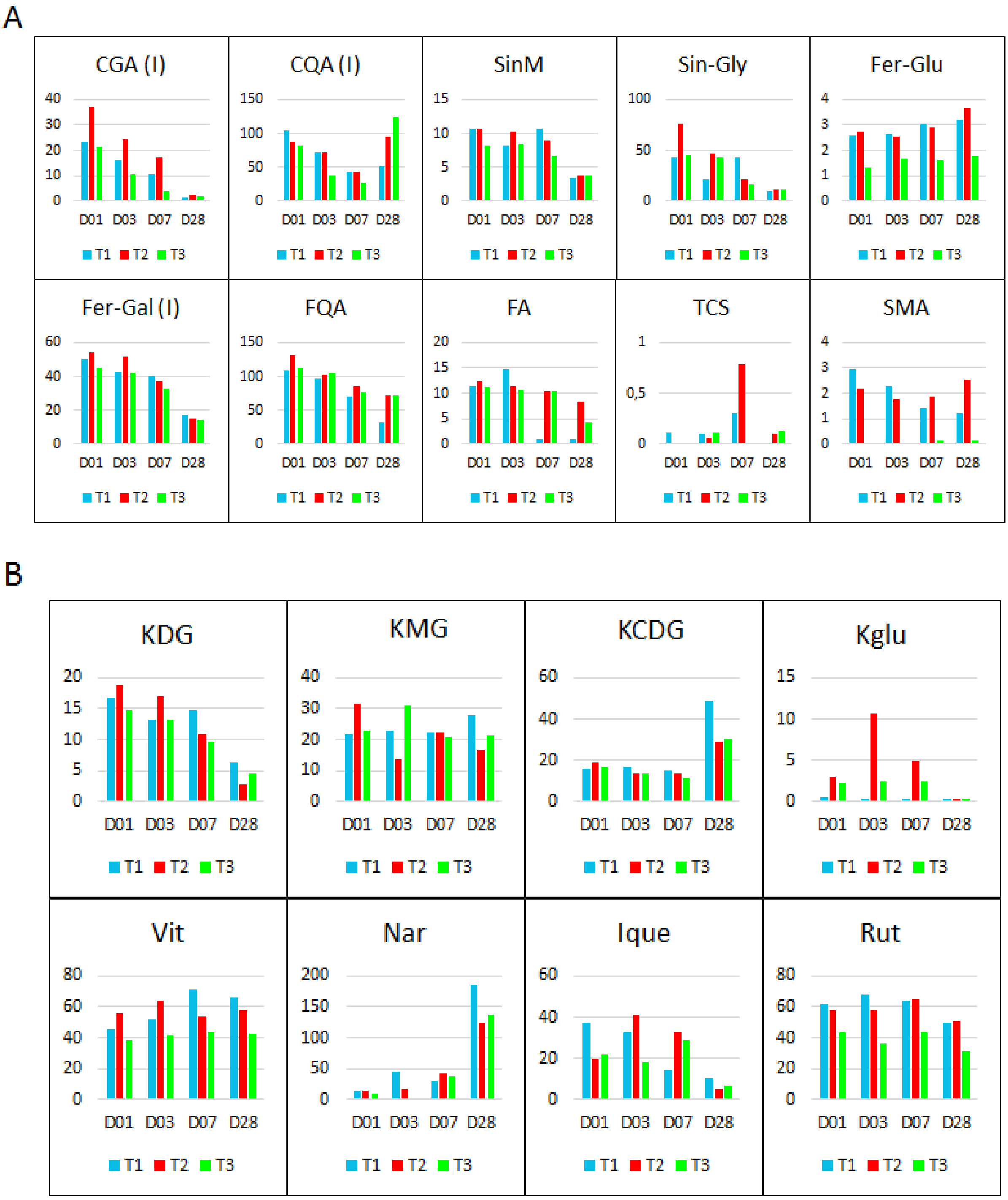

Figure S6: An overview of relative phenolics and polyamine content fluctuations in different treatments. The column graphs were constructed based on integrated peak areas (IPA) of pure and/or conjugated phenolic acids and polyamines (**A**) and flavonoids (**B**) in non-stress- (T1, blue), naïve salt stress- (T2, red), and biostimulant treated salt stress (T3, green) conditions across all time points (days 1, 3, and 28). **Abbreviations**: CouQA, coumaroyl-quinic acid; CouGA, coumaroyl-galactarate; CQA, caffeoyl-quinic acid; CGA, caffeoyl-galactarate; FA, ferulic acid; FQA, feruloyl-quinic acid; Fer-glu, feruloyl-glucoside; Sin-Gly, sinapoyl-glycoside; SinM, sinapoyl-malate; TCS, tricoumaroyl-spermidine, SMA, spermidine monoaldehyde; Que, quercetin; Qtrn, quercetrin; Ique, isoquercetin; Kglu, kaemferol glucoside; KCDG, kaempferol coumaroyl-diglucosyl-glucoside; KMG, kaempferol malonoyl-glucoside; KDG, kaempferol diglucosyl-glucoside; Vit, vitexin; Nar, naringin; Rut, rutin.

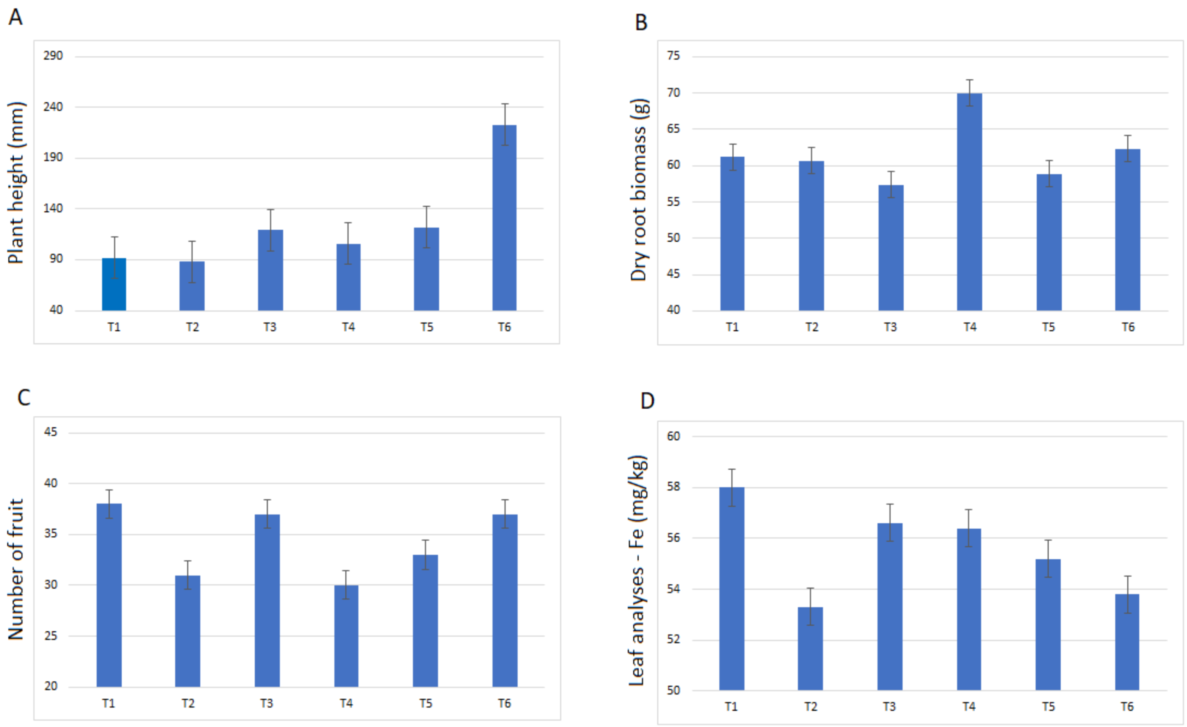

Figure S7: The effects of different treatments on the agronomic and phenotypic states of the plant samples. The graphs present general increases in plant height (**A**), dry root biomass (**B**), number of fruits (**C**), and leaf analyses of iron content (**D**) induced by Si treatment of the salt stressed plants (T2) at both foliar (T4 and T6) and soil (T3 and T5) applied biostimulant.

Table S1. Metamapp data used for generation of a network (Figure 4).

				Salt stress effect		Si biostimulant effect	
**PubChem_ID**	**KEGG_ID**	**SMILES**	**Compound_Name**	**Fold change**	**p-value**	**Fold change**	**p-value**
**699414**	C01494	COC1=CC=C(C=C1)C=CC(=O)O	4-Methoxycinnamic acid	1.00E+00	5.00E-02	1.00E+00	5.00E-02
**70912**	C02710	CC(C)CC(C(=O)O)NC(=O)C	Acetyl leucine	9.17E-01	7.05E-01	6.52E-02	1.20E-06
**700653**	C03137	CC(=O)NC(CC1=CNC2=CC=CC=C21)C(=O)O	Acetyl tryptophan	6.76E-01	1.56E-01	1.54E+00	9.15E-02
**5484352**	C00041	CC(C(=O)NC(C)C(=O)O)N	Alanyl alanine	1.01E+00	5.00E-02	1.00E+02	2.05E-03
**16950**	C03406	C(CC(C(=O)O)N)CN=C(N)NC(CC(=O)O)C(=O)O	Argininosuccinate	1.01E+00	5.00E-02	1.00E+00	5.00E-02
**54670067**	C00072	C(C(C1C(=C(C(=O)O1)O)O)O)O	Ascorbic acid	6.13E-02	3.72E-02	9.15E-01	9.35E-01
**5459979**	C03062	C1=CC(=C(C=C1C=CC(=O)OC(C(C(C(C(=O)O)O)O)O)C(=O)O)O)O	Caffeoylglucaric acid	1.17E+05	3.26E-01	2.66E-01	2.05E-04
**5281761**	C10433	C1=CC(=C(C=C1C=CC(=O)OC2C(C(C(C(O2)CO)O)O)O)O)O	Caffeoylglycoside	4.76E+04	1.22E-04	7.47E-01	1.95E-01
**10155076**	C00852	C1C(C(C(CC1(C(=O)O)OC(=O)C=CC2=CC(=C(C=C2)O)O)O)O)O	Caffeoylquinic acid	1.35E+05	7.92E-03	8.83E-01	1.00E-01
**93072**	C00438	C(C(C(=O)O)NC(=O)N)C(=O)O	Carbamoylaspartate	1.01E+00	5.00E-02	1.00E+00	5.00E-02
**448878**	C00073	CSCCC(C(=O)O)NC(=O)N	Carbamylmethionine	9.06E-01	7.84E-02	9.30E-01	1.69E-01
**12039**	C00251	C=C(C(=O)O)OC1C=C(C=CC1O)C(=O)O	Chorismic acid	1.01E+00	5.00E-02	1.00E+00	5.00E-02
**311**	C00158	C(C(=O)O)C(CC(=O)O)(C(=O)O)O	Citric acid	1.00E+00	5.00E-02	1.00E+00	5.00E-02
**6440013**	C00223	CC(C)(COP(=O)(O)OP(=O)(O)OCC1C(C(C(O1)N2C=NC3=C(N=CN=C32)N)O)OP(=O)(O)O)C(C(=O)NCCC(=O)NCCSC(=O)C=CC4=CC=C(C=C4)O)O	Coumaroylglucaric acid	9.22E-01	5.80E-01	2.78E-01	7.60E-05
**14158103**	C12208	C1C(C(C(CC1(C(=O)O)O)OC(=O)C=CC2=CC=C(C=C2)O)O)O	Coumaroylquinic acid	9.12E-01	5.58E-01	6.83E-01	1.65E-02
**6251**	C00392	C(C(C(C(C(CO)O)O)O)O)O	D-mannitol	1.00E+00	5.00E-02	1.00E+00	5.00E-02
**5281416**	C09263	C1=CC(=O)OC2=CC(=C(C=C21)O)O	Esculetin	1.00E+00	5.00E-02	1.00E+00	5.00E-02
**445858**	C01494	COC1=C(C=CC(=C1)C=CC(=O)O)O	Ferulic acid	1.00E+02	1.35E-02	1.29E+00	5.94E-01
**14104340**	C03156	COC1=C(C=CC(=C1)C=CC(=O)OC(C(C(C(C(=O)O)O)O)O)C(=O)O)O	Feruloylgalactarate	8.23E-01	4.55E-03	5.23E-01	1.50E-06
**13962928**	C17759	COC1=C(C=CC(=C1)C=CC(=O)OC2C(C(C(C(O2)CO)O)O)O)O	Feruloylglucose	8.88E-01	6.68E-02	8.29E-01	3.75E-04
**9799386**	C02572	COC1=C(C=CC(=C1)C=CC(=O)OC2CC(CC(C2O)O)(C(=O)O)O)O	Feruloylquinic acid	8.16E-01	6.94E-02	7.83E-01	4.00E-02
**5273568**	C09266	COC1=C(C(=C2C(=C1)C=CC(=O)O2)OC3C(C(C(C(O3)CO)O)O)O)O	Fraxin	1.00E+00	5.00E-02	1.00E+00	5.00E-02
**33037**	C00818	C(C(C(C(=O)O)O)O)(C(C(=O)O)O)O	Glucaric acid	1.00E+00	5.00E-02	1.00E+00	5.00E-02
**4525487**	C00302	C(CC(=O)[O-])C(C(=O)[O-])[NH3+]	Glutamate	2.03E+05	3.02E-06	1.00E-02	2.66E-16

**5287779**	C00064	CC(C)(C)OC(=O)C(CCC(=O)N)N	Glutamine butyl ester	2.60E+05	4.49E-01	9.87E+01	3.78E-02
**123938**	C00669	C(CC(=O)NC(CS)C(=O)O)C(C(=O)O)N	Glutamylcystine	1.00E+00	5.00E-02	1.00E+00	5.00E-02
**92843**	C02155	CC(C)CC(C(=O)O)NC(=O)CN	Glycylleucine	4.06E+05	2.12E-01	5.10E+00	9.48E-08
**13932172**	C21980	CCCCCC=CCC=CCCCCCC(=O)O	Hexadecadienoic acid	1.07E+05	4.26E-02	2.21E+01	1.23E-01
**22508268**	C04717	CCCCCC(CCCCCCCC=CC=CC(=O)O)OO	Hydroperoxyoctadecadienoic acid	1.86E+05	4.12E-03	1.66E-01	2.92E-06
**5312869**	C22001	CCCCCCCCCCCC(CC(=O)O)O	Hydroxymyristic acid	1.00E+00	5.00E-02	1.00E+00	5.00E-02
**5810**	C01157	C1C(CNC1C(=O)O)O	Hydroxyproline	1.31E+05	5.51E-05	3.50E-03	5.55E-24
**144**	C01017	C1=CC2=C(C=C1O)C(=CN2)CC(C(=O)O)N	Hydroxytryptophan	4.01E+05	1.86E-10	8.07E-02	3.79E-13
**440406**	C04611	C1C(C(C(C(O1)OC2C(C(C(C(C2O)O)OC(=O)CC3=CNC4=CC=CC=C43)O<br>)O)O)O)O	Indolyl acetyl myoinositol arabinoside	1.00E+00	5.00E-02	1.00E+02	1.50E-02
**6306**	C00407	CCC(C)C(C(=O)O)N	Isoleucine	8.94E-01	6.57E-02	5.25E-01	2.29E-03
**5280804**	C05623	C1=CC(=C(C=C1C2=C(C(=O)C3=C(C=C(C=C3O2)O)O)OC4C(C(C(C(O4)<br>CO)O)O)O)O)O	Isoquercitin	1.00E+00	5.00E-02	1.00E+00	5.00E-02
**5280863**	C05903	C1=CC(=CC=C1C2=C(C(=O)C3=C(C=C(C=C3O2)O)O)O)O	Kaempferol	7.63E-01	2.42E-02	1.00E+02	6.53E-06
**583024**	C12634	OC[C@H]1O[C@@H](O[C@H]2[C@H](Oc3c(oc4cc(O)cc(O)c4c3=O)<br>-<br>c3ccc(O)cc3)O[C@H](CO)[C@@H](O)[C@@H]2O)[C@H](O)[C@@H<br>](O)[C@@H]1O	Kaempferol diglucosyl-glucoside	8.79E-01	3.83E-01	7.48E-01	3.34E-02
**5480982**	C12249	C1=CC(=CC=C1C2=C(C(=O)C3=C(C=C(C=C3O2)OC4C(C(C(C(O4)CO)O<br>)O)O)O)O)O	Kaempferol glucoside	1.00E+00	5.00E-02	1.00E+02	1.42E-04
**104152**	C00047	CC(=O)O.C(CCN)CC(C(=O)O)N	Lysine acetate	2.63E+05	2.58E-02	8.71E-02	3.91E-04
**525**	C00149	C(C(C(=O)O)O)C(=O)O	Malic acid	8.72E-01	3.35E-01	2.55E+00	4.45E-10
**442428**	C09789	CC1C(C(C(C(O1)OC2C(C(C(OC2OC3=CC(=C4C(=O)CC(OC4=C3)C5=CC<br>=C(C=C5)O)O)CO)O)O)O)O)O	Naringin	4.23E-01	1.26E-01	1.84E+00	1.27E-01
**445638**	C08362	CCCCCCC=CCCCCCCCC(=O)O	Palmitoleic acid	4.14E+05	2.65E-02	1.89E-01	2.78E-02
**6140**	C02057	C1=CC=C(C=C1)CC(C(=O)O)N	Phenylalanine	9.55E-01	8.85E-01	1.00E-02	6.97E-10
**91493**	C04442	C(C(C(C(C(C(=O)O)O)O)O)O)OP(=O)(O)O	Phosphogluconate	4.22E-01	2.19E-03	6.49E+00	1.12E-11
**115244**	C10172	C[N+]1(CCCC1C(=O)[O-])C	Proline betaine	1.00E+00	5.00E-02	1.00E+02	1.53E-02
**5280343**	C00389	C1=CC(=C(C=C1C2=C(C(=O)C3=C(C=C(C=C3O2)O)O)O)O)O	Quercetin	9.19E-01	2.38E-01	1.11E+00	1.75E-01
**5280459**	C01750	CC1C(C(C(C(O1)OC2=C(OC3=CC(=CC(=C3C2=O)O)O)C4=CC(=C(C=C4<br>)O)O)O)O)O	Quercitrin	1.82E+05	4.15E-06	1.00E+02	6.03E-03
**6508**	C00296	C1C(C(C(CC1(C(=O)O)O)O)O)O	Quinic acid	1.12E+05	6.33E-01	1.29E+00	2.24E-01
**5280805**	C05625	CC1C(C(C(C(O1)OCC2C(C(C(C(O2)OC3=C(OC4=CC(=CC(=C4C3=O)O)<br>O)C5=CC(=C(C=C5)O)O)O)O)O)O)O)O	Rutin	2.70E+05	3.09E-01	2.25E+00	1.30E-01

**338**	C00805	C1=CC=C(C(=C1)C(=O)O)O	Salicylic acid	3.43E+05	2.92E-06	5.48E-01	4.53E-04
**5280665**	C01175	COC1=CC(=CC(=C1O)OC)C=CC(=O)OC2C(C(C(OC2OC(=O)C=CC3=CC(=C(C(=C3)OC)O)OC)CO)O)O	Sinapoylglycoside	5.74E-01	3.08E-06	8.42E-01	1.14E-01
**11953815**	C02887	COC1=CC(=CC(=C1O)OC)C=CC(=O)OC(CC(=O)O)C(=O)O	Sinapoylmalate	7.75E-01	1.82E-01	1.14E-01	7.33E-06
**1103**	C00750	C(CCNCCCN)CNCCCN	Spermine monoaldehyde	1.00E+00	5.00E-02	1.00E+02	7.37E-03
**65576**	C10826	CC1CCC2(C(C3C(O2)CC4C3(CCC5C4CCC6C5(CCC(C6)O)C)C)C)NC1	Tomatidine	1.00E+00	5.00E-02	1.00E+02	6.28E-04
**28523**	C10827	CC1CCC2(C(C3C(O2)CC4C3(CCC5C4CCC6C5(CCC(C6)OC7C(C(C(C(O7)CO)OC8C(C(C(C(O8)CO)O)OC9C(C(C(CO9)O)O)O)OC2C(C(C(C(O2)CO)O)O)O)O)O)C)C)C)NC1	Tomatine	9.10E-01	2.41E-01	8.35E-01	5.72E-03
**14777879**	C18069	C1=CC(=CC=C1C=CC(=O)NCCCCN(CCCNC(=O)C=CC2=CC=C(C=C2)O)C(=O)C=CC3=CC=C(C=C3)O)O	Tricoumaroylspermidine	1.09E+05	8.73E-01	1.00E-02	1.75E-03
**6305**	C00806	C1=CC=C2C(=C1)C(=CN2)CC(C(=O)O)N	Tryptophan	1.10E+05	3.02E-01	8.74E-01	1.71E-01
**15376**	C09251	CCC12CCCN3C1C4=C(CC3)C5=CC=CC=C5N4C(C2)(C(=O)OC)O	Vincamine	1.00E+00	5.00E-02	1.00E+00	5.00E-02
**5280441**	C01460	C1=CC(=CC=C1C2=CC(=O)C3=C(O2)C(=C(C=C3O)O)C4C(C(C(C(O4)CO)O)O)O)O	Vitexin	8.76E-01	3.78E-02	8.61E-01	5.80E-03

Table S2: An overview of all metabolites detected and annotated from all different treatments (T1-T6) at all time -points _days 1, 3, 7 and 28).

Metabolite ID	Abbr.	Rt (min)	*m/z*	Diagnostic Fragment Ions	Adducts	Molecular Formula	Molecular Weight	Metabolite Class
**Malic acid**	MA	0.96	133.01	115	$[M - H]^-$	$C_4H_6O_5$	134.09	Organic acid
**Coumaroyl-glucaric acid**	CouGA	3.51	355.07	257, 220,129		$C_{15}H_{18}O_8$	326	HCA-sugar
**Dehydrotomatine (I)**	DHT (I)	6.3	1031.55	898, 868, 576	$[M - H]^-$	$C_{50}H_{81}NO_{21}$	1032.5	Glycoalkaloid
**Hydroxyproline**	Hpro	4.72	149.10	84,59	$[M-OH]^-$	$C_5H_9NO_3$	131.13	Amino acid
**Salicylic acid**	SA	6.86	137.02	93	$[M - H]^-$	$C_7H_6O_3$	138.12	HCA
**Glutamate**	Glu	6.25	148.10	147, 128	$[M + H]^+$	$C_5H_9O_4$	147.12	Amino acid

**Alanyl-alanine**	AlA	2.16	178.12	86, 91	$[M + H]^+$	$C_6H_{12}N_2O_3$	160.17	Amino acid
**Acetyl-leucine**	AL	4.9	174.10	86	$[M + H]^+$	$C_8H_{15}NO_3$	173.21	Amino acid
**Ascorbic acid**	AA	5.48	175.02	147, 87, 69	$[M - H]^-$	$C_6H_8O_6$	176.12	Organic acid
**Carbamoyl-aspartate**	CLA	5	177.05	134	$[M + H]^+$	$C_5H_8N_2O_5$	176.13	Amino acid
**Glycyl-leucine**	GL	5.4	189.12	86, 55, 30	$[M + H]^+$	$C_8H_{16}N_2O_3$	188.22	Amino acid
**Quinic acid**	QA	4.4	191.05	175, 156, 146	$[M - H]^-$	$C_7H_{12}O_6$	192.17	Organic acid
**Carbamyl-methionine**	CAM	3.74	191.05	150	$[M + H]^+$	$C_6H_{12}N_2O_3S$	192.24	Amino acid
**Ferulic acid**	FA	6.96	193.05	178, 149, 134	$[M – H]^-$	$C_{10}H_{10}O_4$	194.18	HCA
**Glutamine butyl ester**	GTBE	6.16	220.17	156, 128	$[M – H]^-$	$C_9H_{18}N_2O_3$	202.25	Amino acid
**Ganic Lysine acetate**	LysA	4.71	207.13		$[M + H]^+$	$C_8H_{18}N_2O_4$	206.24	Amino acid
**Hydroxytryptophan**	Htrp	1.56	219.08	202, 160, 132	$[M - H]^-$	$C_{11}H_{12}N_2O_3$	220.22	Amino acid
**Chorismic acid**	CHA	5.37	225.04	189, 171	$[M - H]^-$	$C_{10}H_{10}O_6$	226.18	Organic acid
**Hydroxymyristic acid**	HmyrA	12.14	265.18	227	$[M\text{-}OH]^-$	$C_{14}H_{28}O_3$	244.37	Organic acid
**Acetyl-tryptophan**	Atrp	5.75	245.09	216, 128, 98	$[M - H]^-$	$C_{13}H_{14}N_2O_3$	246.26	Amino acid
**Glutamyl-cystine**	BGGC	8.45	499.12	128, 120, 82	$[M\text{-}H]^-$	$C_8H_{14}N_2O_5S$	250.3	Amino acid
**Hexadecadienoic acid**	HDDA	8.74	275.20		$[M + Na]^+$	$C_{16}H_{28}O_2$	252.39	Fatty acid
**Palmitoleic acid**	PA	13.54	255.20	237, 219, 171	$[M + H]^+$	$C_{16}H_{30}O_2$	254.41	Fatty acid
**Phosphogluconate**	Pglu	1.04	275.02	256, 176, 96	$[M - H]^-$	$C_6H_{13}O_{10}P$	276.14	Sugar
**Quercetin**	Que	5.82	301.05	284, 255, 227	$[M - H]^-$	$C_{21}H_{20}O_{11}$	302.24	Flavonoid

**Hydroperoxyoctadecadienoic acid**	HPDA	12.9	311.22	206	[M - H]$^-$	$C_{18}H_{32}O_4$	312.4	Fatty acid
**Coumaroyl-quinic acid (I)**	CouQA (I)	4.81	337.09	337, 163	[M - H]$^-$	$C_{16}H_{18}O_8$	338.31	HCA
**Coumaroyl-quinic acid (II)**	CouQA (II)	4.59	337.09	337, 163, 145	[M - H]$^-$	$C_{16}H_{18}O_8$	338.32	HCA
**Sinapoyl-malate**	SinM	2.75	385.08	223, 164, 149	[M - H]$^-$	$C_{15}H_{16}O_9$	340.28	HCA-sugar
**Caffeoyl-glycoside**	Cgly	2.99	341.09	179, 135	[M - H]$^-$	$C_{15}H_{18}O_9$	342.3	HCA-sugar
**Feruloyl-quinic acid**	FQA	4.93	367.10	193, 191	[M - H]$^-$	$C_{17}H_{20}O_9$	368.3	HCA
**Tomatidine**	Tomd	7.37	416.35	398, 255	[M + H]$^+$	$C_{27}H_{45}NO_2$	415.7	Glycoalkaloid
**Kaempferol glucoside**	Kglu	6.38	447.09	284, 255, 227	[M - H]$^-$	$C_{21}H_{20}O_{11}$	448.4	Flavonoid-sugar
**Quercitrin**	Que	5.54	449.11	471, 449, 303	[M + H]$^+$	$C_{21}H_{20}O_{11}$	448.4	Flavonoid
**Indolyl-acetyl-myo-inositol arabinoside**	IMIA	1.91	538.15		[M + HCOONa$^+$	$C_{21}H_{27}NO_{11}$	469.4	Sugar
**Tri-coumaroyl-spermidine**	TCS	8.29	584.27		[M + H]$^+$	$C_{34}H_{37}N_3O_6$	583.7	Polyamine
**Rutin**	Rut	5.59	609.04	300.028, 271,025, 255,030, 151.002	[M - H]$^-$	$C_{27}H_{30}O_{16}$	610.5	Flavonoid
**Isoleucine**	Ile	1.42	132.10	86	[M + H]$^+$	$C_6H_{13}NO_2$	131.17	Amino acid
**Caffeoyl-quinic acid**	CQA (II)	3.8	353.09	191	[M - H]$^-$	$C_{16}H_{18}O_9$	354.31	HCA
**Feruloyl-glucose**	Fer-Glu	4.28	355.10	193	[M - H]$^-$	$C_{16}H_{20}O_9$	356.32	HCA-sugar
**Isoquercitin**	Ique	5.64	465.1	303	[M - H]$^-$	$C_{21}H_{20}O_{12}$	464.1	Flavonoid
**Phenylalanine**	Phe	1.97	166.08	120 103	[M + H]$^+$	$C_9H_{11}NO_2$	165.19	Amino acids

**Caffeoyl-quinic acid (II)**	CQA (III)	4.4	353.08	191 173 135	$[M - H]^-$	$C_{16}H_{18}O_9$	354.31	HCA
**Sinapoyl-glycoside**	Sin-Gly	4.27	385.11	223 205 179	$[M - H]^-$	$C_{17}H_{22}O_{10}$	386.3	HCA-sugar
**Caffeoyl-quinic acid (IV)**	CQA (IV)	4.57	353.05	191 179 173 135	$[M - H]^-$	$C_{16}H_{18}O_9$	354.31	HCA
**Caffeoyl-quinic acid (I)**	CQA (I)	2.72	353.86	191 179 173 135	$[M - H]^-$	$C_{16}H_{18}O_9$	354.32	HCA
**Caffeoyl-glucaric acid (I)**	CGA (I)	1.98	371.06	209 173 164 129	$[M - H]^-$	$C_{15}H_{16}O_{11}$	354.33	HCA-sugar
**Feruloyl-galactarate (I)**	Fer-gal (I)	3.08	385.07	223 191 193 189	$[M - H]^-$	$C_{16}H_{18}O_{11}$	356.534	HCA-sugar
**Feruloyl-galactarate (II)**	Fer-gal (II)	3.95	385.09	223 191 193 189	$[M - H]^-$	$C_{16}H_{18}O_{11}$	356.535	HCA-sugar
**Vitexin**	Vit	5.71	431.09	341 311 283 269	$[M - H]^-$	$C_{21}H_{20}O_{10}$	432.38	Flavonoid
**Tryptophan**	Trp	2.97	205.09	116, 142, 158	$[M + H]^+$	$C_{11}H_{12}N_2O_2$	204.23	Amino acid
**Citric acid**	CA	1.22	191.017	173,129,111,85	$[M - H]^-$	$C_6H_8O_7$	192.12	Organic acid
**Caffeoyl-glucaric acid (III)**	CGA (III)	1.52	371.06	209,173,129,85	$[M - H]^-$	$C_{15}H_{16}O_{11}$	372.28	HCA-sugar
**Caffeoyl-glucaric acid (II)**	CGA (II)	1.75	371.06	209,173,143,129,85	$[M - H]^-$	$C_{15}H_{16}O_{11}$	372.29	HCA-sugar
**Kaempferol malonoyl-glucoside (I)**	KMG (I)	4.24	533.09	285, 489	$[M - H]^-$	$C_{24}H_{22}O_{14}$	534.4	Flavonoid-sugar
**Kaempferol malonoyl-glucoside (II)**	KMG (II)	4.07	533.09	285, 490	$[M - H]^-$	$C_{24}H_{22}O_{14}$	534.4	Flavonoid-sugar
**Argininosuccinate**	Arg-succ	4.05	289.12		$[M - H]^-$	$C_{10}H_{18}N_4O_6$	290.27	Amino acid
**Naringin**	Nar	5.77	579.17	271, 151	$[M - H]^-$	$C_{27}H_{32}O_{14}$	580.54	Flavonoid

**Kaempferol diglucosyl-glucoside**	KDG	3.88	771.20		$[M - H]^-$	$C_{33}H_{40}O_{21}$	773.19	Flavonoid-sugar
**Kaempferol coumaroyl-diglucosyl-glucoside**	KCDG	6.52	917.24		$[M - H]^-$	$C_{42}H_{46}O_{23}$	918.25	Flavonoid-sugar
**Dehydrotomatine (II)**	DHT (II)	7.22	1076.50		$[M + HCOOH]^+$	$C_{50}H_{81}NO_{21}$	1032.5	Glycoalkaloid
**Tomatine**	Tom	7.39	1078.55	1034, 1016	$[M + HCOOH]^+$	$C_{50}H_{83}NO_{21}$	1034.19	Glycoalkaloid
**Proline betaine**	PB	1.91	166.08	84	$[M + Na]^+$	$C_7H_{13}NO_2$	143.18	Amino acid
**Spermine monoaldehyde**	SMA	1.93	328.14		$[M + NaCl\_HCOONa]^+$	$C_{10}H_{23}N_3O$	201.26	Polyamine

Table S3: The most significantly altered pathways based on impact factor > 0.1, Holm adjusted *p*-value < 0.05 and FDR < 0.05. The indication letters correspond to the labelled pathways on the pathway impact plot (**Figure 3B**)

	**Pathways**	**Total**	**Hits**	**Holm *p*-value**	**FDR**	**Impact**	**Metabolites (Abbreviations)[1]**
**Significant pathways based on impact factor**							
**E**	Biosynthesis of secondary metabolites	5	4	1.44×10^{-3}	3.88×10^{-4}	1	CouA, C-CoA, Cou-CoA, F-CoA
**F**	Linoleic acid metabolism	4	1	1	1	1	Linoleate
**D**	Flavone and flavonol biosynthesis	10	5	2.51×10^{-3}	5.25×10^{-4}	0.85	Kae, Que, Qtrn, Kae-glu, Que-glu
**C**	Stilbenoid biosynthesis	8	5	6.2×10^{-4}	3.12×10^{-4}	0.735	Cou-CoA, Cou-SKA, CGA, C-CoA, F-CoA
**B**	Flavonoid biosynthesis	47	10	1.5×10^{-3}	3.88×10^{-4}	0.517	Cou-SKA, Cou-CoA, CGA, Kae, D-kae, C-CoA, Nar, F-CoA, Que
**L**	Phenylalanine metabolism	11	1	1	1	0.471	Phe

[1] CouA, Coumaric acid; C-CoA, Caffeoyl-CoA; Cou-CoA, Coumaroyl-CoA; F-CoA, Feruloyl-CoA; Kae, Kaempferol; Que, Quercetin; Qtrn, Quercetrin; glu, glucoside; SKA, shikimate; Nar, Naringin; Phe, Phenylalanine; Trp, Tryptophan; tyr, tyrosine; CHA, chorismic acid; AA, Ascobic acid; GA, galactarate; Arg-Succ, arginino-succinate; Gln, Glutamine; Asp, Aspartate; MA, Malic acid; CA, Citric acid; FA, Ferulic acid; CQA, Caffeoyl-quinic acid; CGA, Chlorogenic acid; SinM, Sinapoy- malate; Sin-gly, Sinapoyl-glycoside.

**A**	Phenylpropanoid biosynthesis	46	12	1.7×10^{-5}	1.74×10^{-5}	0.389	FA, CQA, Cou-CoA, CGA, Phe, CouA, C-CoA, F-CoA, Sin-gly, TCS, SinM, F-CoA
**I**	Phe, tyr and trp biosynthesis	22	3	1	1.542×10^{-1}	0.228	CHA, Trp, Phe
**K**	Ascorbate and aldarate metabolism	18	2	1	1	0.216	AA, GA
**H**	Ala, asp and glu metabolism	22	4	1	1.542×10^{-1}	0.212	Arg-succ, Ala, Gln, Asp
**M**	Citrate cycle (TCA cycle)	20	2	1	1	0.149	MA, CA
**Significant pathways based on FDR and P-value**							
**A**	Phenylpropanoid biosynthesis	46	12	1.7E-05	1.74×10^{-5}	0.389	FA, CQA, Cou-CoA, CGA, Phe, CouA, C-CoA, F-CoA, Sin-gly, TCS, SinM, F-CoA
**C**	Stilbenoid biosynthesis	8	5	6.2×10^{-4}	3.12×10^{-4}	0.735	Cou-CoA, Cou-SKA, CGA, C-CoA, F-CoA
**E**	Biosynthesis of secondary metabolites -	5	4	1.44×10^{-3}	3.88×10^{-4}	1	CouA, C-CoA, Cou-CoA, F-CoA
**B**	Flavonoid biosynthesis	47	10	1.5×10^{-3}	3.88×10^{-4}	0.517	Cou-SKA, Cou-CoA, CGA, Kae, D-kae, C-CoA, Nar, F-CoA, Que
**D**	Flavone and flavonol biosynthesis	10	5	2.51×10^{-3}	5.25×10^{-4}	0.85	Kae, Que, Qtrn, Kae-glu, Que-glu
**G**	Aminoacyl-tRNA biosynthesis	46	6	2.567×10^{-1}	4.513×10^{-2}	0	Phe, Arg, Gln, Asp, Met, Ala, Ile, Leu, Thr, Trp
